# Distinct immune signatures discriminate between asymptomatic and presymptomatic SARS-CoV-2^pos^ subjects

**DOI:** 10.1038/s41422-021-00562-1

**Published:** 2021-09-24

**Authors:** Shanhe Yu, Caixia Di, Shijun Chen, Mingquan Guo, Jiayang Yan, Zhaoqin Zhu, Li Liu, Ruixue Feng, Yinyin Xie, Ruihong Zhang, Juan Chen, Mengxi Wang, Dong Wei, Hai Fang, Tong Yin, Jinyan Huang, Saijuan Chen, Hongzhou Lu, Jiang Zhu, Jieming Qu

**Affiliations:** 1 Shanghai Institute of Hematology, State Key Laboratory of Medical Genomics, National Research Center for Translational Medicine at Shanghai, Collaborative Innovation Center of Hematology, Ruijin Hospital affiliated to Shanghai Jiao-Tong University School of Medicine, Shanghai, China; 2 Department of Respiratory and Critical Care Medicine, Ruijin Hospital, Institute of Respiratory Diseases, School of Medicine, Shanghai Jiao-Tong University, Shanghai, China; 3 Key Laboratory of Emergency Prevention, Diagnosis and Treatment of Respiratory Infectious Diseases, Shanghai, China; 4 Department of Laboratory Medicine, Shanghai Public Health Clinical Center, Fudan University, Shanghai, China; 5 Department of Infectious Diseases and Immunology, Shanghai Public Health Clinical Center, Fudan University, Shanghai, China; 6 Department of Infectious Disease, Research Laboratory of Clinical Virology, Ruijin Hospital, School of Medicine, Shanghai Jiao-Tong University, Shanghai, China; 7 National Research Center for Translational Medicine at Shanghai, Shanghai, China

**Keywords:** Mechanisms of disease, Immunology

## Abstract

Increasing numbers of SARS-CoV-2-positive (SARS-CoV-2^pos^) subjects are detected at silent SARS-CoV-2 infection stage (SSIS). Yet, SSIS represents a poorly examined time-window wherein unknown immunity patterns may contribute to the fate determination towards persistently asymptomatic or overt disease. Here, we retrieved blood samples from 19 asymptomatic and 12 presymptomatic SARS-CoV-2^pos^ subjects, 47 age/gender-matched patients with mild or moderate COVID-19 and 27 normal subjects, and interrogated them with combined assays of 44-plex CyTOF, RNA-seq and Olink. Notably, both asymptomatic and presymptomatic subjects exhibited numerous readily detectable immunological alterations, while certain parameters including more severely decreased frequencies of CD107a^low^ classical monocytes, intermediate monocytes, non-classical monocytes and CD62L^hi^ CD8^+^ T_naïve_ cells, reduced plasma STC1 level but an increased frequency of CD4^+^ NKT cells combined to distinguish the latter. Intercorrelation analyses revealed a particular presymptomatic immunotype mainly manifesting as monocytic overactivation and differentiation blockage, a likely lymphocyte exhaustion and immunosuppression, yielding mechanistic insights into SSIS fate determination, which could potentially improve SARS-CoV-2 management.

## Introduction

The pandemic coronavirus disease 2019 (COVID-19) caused by severe acute respiratory syndrome coronavirus 2 (SARS-CoV-2) infection has become an unprecedented world-wide emergency lasting for over one year, with 185,291,530 global cases including 4,010,834 deaths having been confirmed until 9 July 2021^1,2^ (https://covid19.who.int). COVID-19 patients present with a wide spectrum of illness severity from mild through moderate diseases to the severe situations necessitating intensive care. Of note, with the availability of RT-PCR kit assaying viral RNA of the nasopharyngeal swabs, more and more seemingly healthy persons at the so-called silent SARS-CoV-2 infection stage (SSIS) are being tested positive for SARS-CoV-2 (SARS-CoV-2^pos^). Longitudinal studies showed that approximately three quarters of them virtually were the real asymptomatic cases who, until their SARS-CoV-2 test turning to negative, manifested neither COVID-19-related symptoms nor CT-confirmed pneumonia with or without receiving nonspecific medication,^3^ leaving the rest being presymptomatic cases that would progress into the acute phase. Recent epidemiologic studies indicated that asymptomatic and presymptomatic transmissions at SSIS together accounted for 75.9% of the total SARS-CoV-2 transmission,^4^ with the presymptomatic subjects being more infectious.^5^ However, viral detection assay alone is unable to distinguish the presymptomatic cases from the asymptomatic subjects.^3^

SARS-CoV-2 infection in human beings causes a broad range of immunological alterations, which might exert either protective or pathogenic effects to influence the disease trajectories,^6^ and the age, gender and certain comorbidities, as three well-documented major clinical factors, influence the clinical course of COVID-19 patients probably through affecting discrete SARS-CoV-2-responsive immunity patterns.^7^ A lot of efforts have been devoted to characterizing the particular immunological responses underlying progression of the acute phase. For examples, the severe situations have been shown to be associate with an abnormal monocytic and/or macrophage activation, a stimulated emergency myelopoiesis, and aberrant activation of T and B lymphocytes that probably led to exhaustion at certain functional aspects.^8^ However, very little is known about the immunological responses responsible for the initial disease progression during the SSIS,^9^ and only a serum cytokine profiling of the asymptomatic subjects as compared to that of the symptomatic cases taken within the acute phase was described.^10^ In this study, we sought to determine what specific alterations in immune responsive elements at both cellular and molecular levels are correlated with the opposite fate determination of the SARS-CoV-2^pos^ subjects at the SSIS. We anticipate that the comprehension of this phenomenon will yield insights into identifying particular protective and pathogenic immune mechanisms concerning SARS-CoV-2 propagation, thus facilitating the proper handling of quickly increased populations of SARS-CoV-2^pos^ subjects manifesting no symptoms.

## Results

### Clinical and general immunological features of SARS-CoV-2^pos^ subjects at the SSIS

**Fig. 1:**
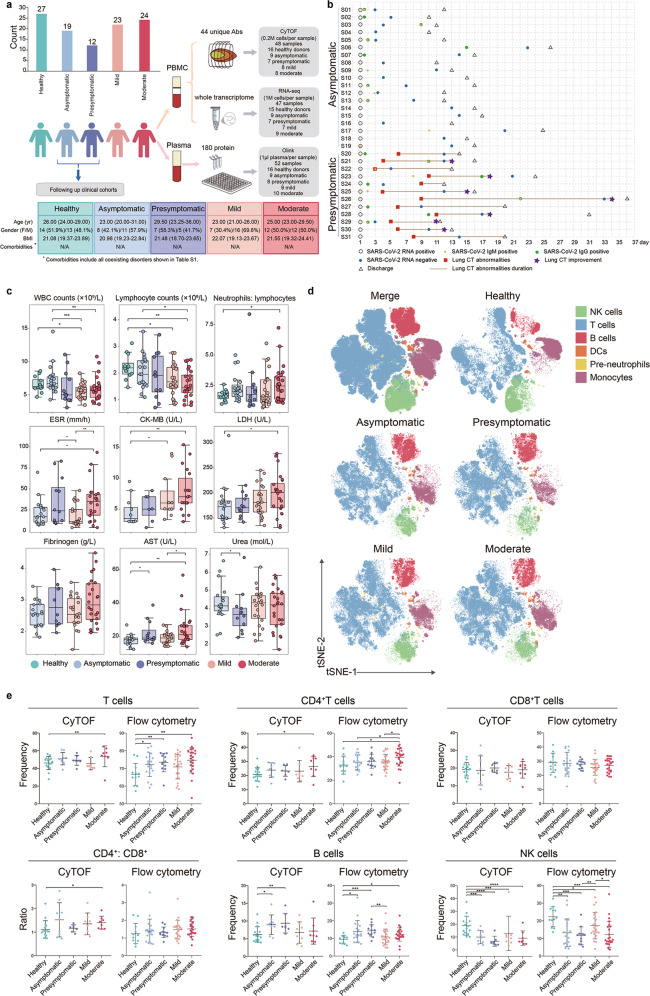
Study design and major clinical parameters of five participant groups. **a** Overview of the study strategy. A cohort including 27 healthy controls, 19 asymptomatic subjects, 12 presymptomatic cases, and 47 mild to moderate COVID-19 patients was enrolled. The individual PBMC samples were profiled by CyTOF and RNA-seq in parallel, and the corresponding plasma samples were analyzed by Olink proteomics assay. **b** The summarized clinical histories of the asymptomatic and presymptomatic subjects. **c** Quantification of the key clinical parameters across the groups. **d** t-SNE plot of CD45^+^ circulating leukocytes, down-sample to 10,000 cells in each sample, based on 38 surface markers of CyTOF analysis. Cells are colored according to major lineage subtypes. **e** The lymphocyte compositions of PBMCs or red blood cell-lysed leukocytes across the groups were measured by CyTOF or flow cytometry. Significance was determined by unpaired Wilcoxon test. *P < 0.05, **P < 0.01, ***P < 0.001, ****P < 0.0001.

We enrolled 31 SARS-CoV-2^pos^ young adults at their SSIS (19 turned out to be persistently asymptomatic subjects as judged by follow-up for > 21 days, and 12 were at presymptomatic stage that later developed into moderate COVID-19), 47 COVID-19 patients at acute phase (23 mild and 24 moderate cases) and 27 healthy donors, and employed combined assays of mass cytometry (CyTOF), RNA sequencing (RNA-seq) and Olink to systemically profile the alterations occurring to their peripheral blood mononuclear cells (PBMCs) and plasma proteins sampled before treatment (Fig. 1a, b and Supplementary information, Table S1). To condense the essential immunological alterations responding to SARS-CoV-2 infection itself, only young adults without comorbidities, but with similar gender distribution, body mass index (BMI), race and habits of smoking and alcohol consumption were selected (Fig. 1a and Supplementary information, Tables S1 and S2). The median duration of viral shedding in the presymptomatic group was longer than that of the asymptomatic group, indicating a shorter viral propagation course for most of the asymptomatic subjects. Nonetheless, a higher proportion of the presymptomatic cases were tested positive for serum SARS-CoV-2 spike protein-specific IgM and/or IgG (Fig. 1b and Supplementary information, Fig. S1a). A detailed blood examination results were documented in the Supplementary information, Tables S3–S5. Consistent with the previous findings,^11^ a reduction in white blood cell (WBC) counts mostly attributed to the lymphopenia was seen in the mild and moderate cases but not in the two SSIS groups, with a significantly increased neutrophils to lymphocytes ratio (NLR) only detected in the moderate cases compared to the healthy persons (Fig. 1c). Interestingly, of several previously reported COVID-19 severity-related serum parameters,^12^ aspartate aminotransferase (AST) value was significantly higher in the presymptomatic group than in the asymptomatic group while the urea value appeared in an inverse way (Fig. 1c and Supplementary information, Tables S5 and S6).

We characterized alterations of the major immune lineages of the PBMCs by CyTOF using 44 antibodies (38 surface markers and 6 cytokines) (Supplementary information, Table S7), and applied the FlowSOM clustering algorithm and dimensionality reduction algorithm t-distributed stochastic neighbor embedding (t-SNE) analysis to reveal distinct clustering of main immune cell types across the cohorts (Fig. 1d). To evaluate the accuracy of CyTOF, we measured the same set of blood samples with ordinary flow cytometry, and found that the lymphocyte frequencies among PBMCs measured independently by these two methods were highly comparable (Supplementary information, Fig. S1b, c). Probably due to the fact that a higher NLR existed in the whole white blood cells of the moderate cases than in other groups (Fig. 1c), T lymphocyte frequency among the neutrophils-depleted PBMCs was slightly elevated in the moderate group (Fig. 1e). Nevertheless, both assays indicated that B cell frequency was seemingly elevated in the two SSIS groups, whereas NK cell frequency dropped early in the SSIS similar to that observed in the acute phase.

### Distinctive alterations of circulating lymphocytes are associated with the asymptomatic and presymptomatic cases

**Fig. 2:**
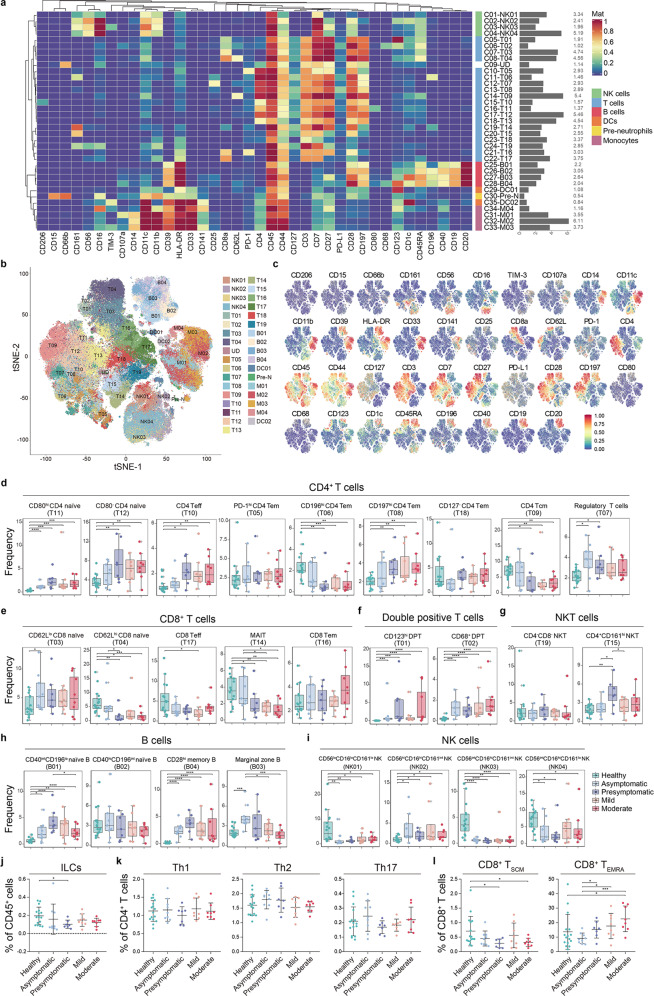
CyTOF analysis of the lymphocytic compositions across groups. **a** Heatmap showing normalized expression of 38 surface markers for 35 clusters identified with PhenoGraph. Relative frequencies are displayed as a bar graph to the right. **b** t-SNE map displaying immune cells pooled from 10,000 cellular events in each individual sample. Cells are colored by PhenoGraph cluster. **c** Normalized expression of indicated markers on the t-SNE map. **d**–**i** Boxplots showing the frequencies of the indicated CD4^+^ T cell clusters (**d**), CD8^+^ T cell clusters (**e**), double-positive T cell clusters (**f**), NKT cell clusters (**g**), B cell clusters (**h**) and NK cell clusters (**i**) across the groups. **j**–**l** Scatter plots showing the frequencies of the ILCs within CD45^+^ cells (**j**), the Th1, Th2 and Th17 within CD4^+^ T cells (**k**), CD8^+^ T_SCM_ and CD8^+^ T_EMRA_ within CD8^+^ T cells (**l**) by manual gating across the groups. Significance was determined by unpaired Wilcoxon test. *P < 0.05, **P < 0.01, ***P < 0.001, ****P < 0.0001.

In-depth analyses of the 38 surface marker expression profile of immune cell clusters were visualized in a heatmap (Fig. 2a), with cluster identities and heterogeneity in marker level shown in single-cell level by t-SNE (Fig. 2b, c and Supplementary information, Fig. S2a). This approach identified 35 clusters including four NK subsets, 19 CD3^+^ T cell subsets, four B cell subsets, two dendritic cell (DC) subsets, four monocytic subsets and one pre-neutrophil subset (Fig. 2a, b and Supplementary information, Table S8). Principle components analysis (PCA) based on the variation of cluster frequencies clearly separated the healthy controls from the SARS-CoV-2^pos^ subjects but not different SARS-CoV-2^pos^ groups from each other (Supplementary information, Fig. S2b). This high-degree PCA overlapping concerning the four SARS-CoV-2^pos^ groups, taken with the aforementioned observation that SARS-CoV-2 specific antibodies were detected in approximately 2/3 of subjects at SSIS (Supplementary information, Fig. S1a), highly suggested that certain profound adaptive immunity alterations already took place during the SSIS. Overall, in seven CD4^+^ T cell subsets (Fig. 2d), three CD8^+^ T cell subsets (Fig. 2e), two CD4^+^ CD8^+^ T cell subsets (Fig. 2f), one NKT cell subset (Fig. 2g), and three B cell subsets (Fig. 2h), their frequencies significantly altered at least in one SSIS group compared to those of the healthy controls, which were in the same direction to those observed in the acute phase. Particularly, elevated frequencies of the CD80^–^ naïve CD4^+^ T cell subset (T12), the effector CD4^+^ T cells (T10) and the CD197^hi^ effector memory CD4^+^ T cells (T08) were evident in the presymptomatic group and mild/moderate cases compared with normal subjects, but were less increased or unchanged in the asymptomatic group (Fig. 2d). The frequencies of CD196^hi^ effector memory CD4^+^ T cells (T06) and central memory CD4^+^ T cells (T09) were significantly decreased in the SARS-CoV-2^pos^ participants except for the asymptomatic group (Fig. 2d). Interestingly, Treg cells (T07) was specifically enriched at the SSIS (Fig. 2d). Moreover, CD62L^hi^ naïve CD8^+^ T cell (CD8^+^ T_naïve_ cell, T04) and mucosal-associated invariant T (MAIT, T14) cell frequencies were reduced in all the SARS-CoV-2^pos^ groups except for the asymptomatic group (Fig. 2e). As for B cells, the frequencies of CD40^int^CD196^lo^ naïve B cells (B01) and CD28^hi^ memory B cells (B04) were increased in four SARS-CoV-2^pos^ groups (Fig. 2h). As seen in the mild or moderate cases, the expression levels of all eight lymphocytic functional activation-related markers such as CD44, PD-1 and IFN-γ were significantly altered in at least one lymphocytic population of T and B cells at the SSIS (Supplementary information, Fig. S2c–f). Taken together, these observations indicated the existence of the broadly altered SSIS-related adaptive immunity in response to SARS-CoV-2 infection. Besides, except an elevated cytokine-secreting CD56^hi^ NK subset (NK02), the frequencies of three CD56^lo^/CD56^int^ cytotoxic NK subsets similarly decreased in the SSIS and mild/moderate cases compared to the healthy controls (Fig. 2i).

In search for differential alterations between the asymptomatic and presymptomatic cases, we noticed reduced frequencies of the CD62L^hi^ CD8^+^ T_naïve_ (T04) but an increased frequency of the CD4^+^ CD161^hi^ NKT cell (T15) in the presymptomatic group (Fig. 2e, g). Meanwhile, although there was no statistical difference, a decreasing tendency in the frequency of MAIT (T14) was observed in the presymptomatic group (Fig. 2e). In this regard, the reduction of CD8^+^ T_naïve_ cells or MAIT probably due to their abnormal overactivation was shown to be positively associated with COVID-19 severity.^13,14^ CD4^+^ NKT cell has been shown to promote a Treg-like immunosuppressive activity although its connection with COVID-19 was unknown.^15,16^ Moreover, an enhanced expression of CD44, generally regarded as an activation marker of lymphocytes but a reduced expression of PD-1 in the T cells, B cells and NK cells, occurred selectively to the asymptomatic group (Supplementary information, Fig. S2c–f), probably implicating a balancing response to counteract the SARS-CoV-2-caused overactivation. A specifically elevated frequency of marginal zone B (MZB) cells (B03) that produced innate antibodies recognizing certain PAMPs of infectious agents^17^ likely occurred to the asymptomatic group (Fig. 2h). Taken together, sustained CD8^+^ T_naïve_ cells (T04) and MAIT (T14) frequencies, an elevated MZB subset (B03), an unexpanded NKT subset (T15), and a particular lymphocyte activation pattern as proved by upregulated CD44 but reduced PD-1 marked the asymptomatic group and might contribute to avoidance of immune exhaustion but constitution of protective immunity post SARS-CoV-2 infection, although further clinical investigation and functional experiments are required to verify this assumption.

We further used manual gating to identify specific immune cell subsets of interest and estimated plausible changes in the frequencies of innate or adaptive lymphocytic subsets across the cohorts. Interestingly, the frequency of innate lymphoid cells (ILCs) in the presymptomatic but not asymptomatic subjects was significantly decreased as compared to the normal subjects (Fig. 2j and Supplementary information, Fig. S3a). Nevertheless, the frequencies of T helper 1 (Th1), Th2 and Th17 within CD4^+^ T cells were not significantly different across the cohorts (Fig. 2k and Supplementary information, Fig. S3a). On the other hand, as differentiated by PD-1 expression level within the naïve CD8^+^ T cells,^18^ approximately 1% of them might belong to the so-called stem-like memory T cells (T_SCM_) (Supplementary information, Fig. S3b), and we found that the frequency of CD8^+^ T_SCM_ within CD8^+^ T cells was similarly decreased in the presymptomatic and moderate COVID-19 groups but not in the asymptomatic and mild COVID-19 groups, as compared to the healthy controls (Fig. 2l). Besides, the frequency of terminally differentiated effector memory CD8^+^ T_EMRA_ within CD8^+^ T cells was significantly increased in the presymptomatic, mild and moderate COVID-19 cases as compared to the asymptomatic groups (Fig. 2l and Supplementary information, Fig. S3b).^19^ Taken together, these results indicated a plausible T cell exhaustion in the presymptomatic group rather than in the asymptomatic group.

### Particular monocytic activation pattern and differentiation retardation mark the presymptomatic group

**Fig. 3:**
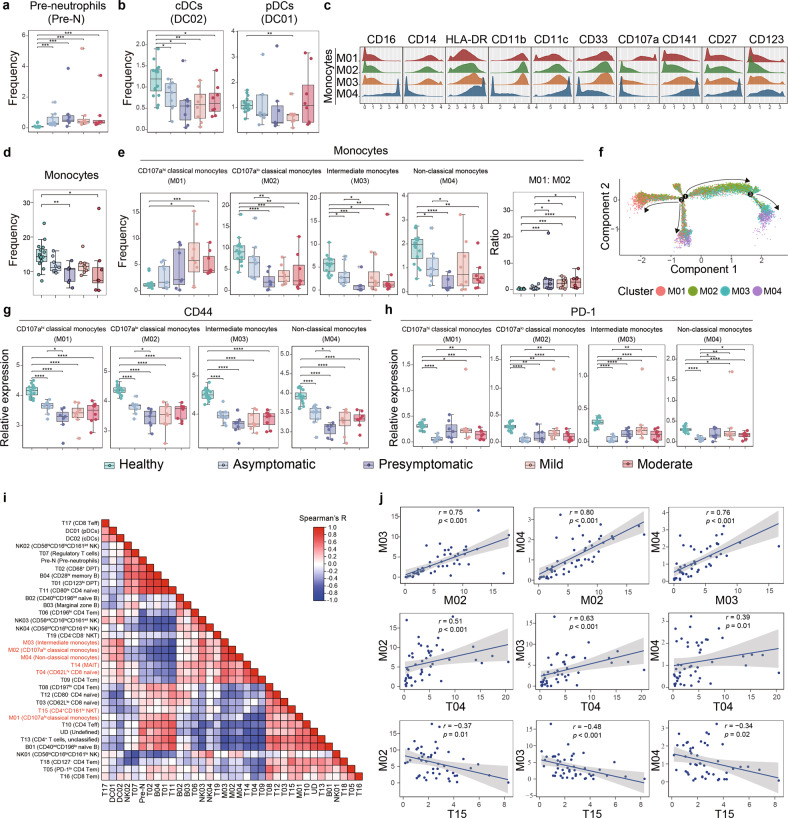
Monocytic abnormalities associate with lymphocytic alterations across the groups. **a**, **b** Boxplots showing the frequencies of the pre-neutrophils (**a**) and those of the cDCs and pDCs (**b**) across the groups. **c** Histograms indicating the expressions of indicated monocytic lineage markers and costimulatory molecules in four monocytic clusters. **d** Boxplot of the frequencies of the total monocytes across the groups. **e** Boxplots showing the frequencies of the monocytic clusters and the cluster M01 to cluster M02 ratios across the groups. **f** Monocle 2 trajectory analysis was performed on a combined dataset of monocytes across the groups. Monocle plot displays monocytes color-coded by different monocytic clusters. **g**, **h** Boxplots showing the expression of CD44 (**g**) and PD-1 (**h**) in the monocytic clusters across the groups. **i** Heatmap showing Spearman correlations using the frequencies of immune cell clusters in all samples. **j** Scatterplots showing relationships between the frequencies of selected clusters. For significant correlations, linear models are shown as blue lines. Significance in **a**, **b**, **d**, **e**, **g**, **h** was determined by unpaired Wilcoxon test. *P < 0.05, **P < 0.01, ***P < 0.001, ****P < 0.0001.

The circulating myeloid cells constitute the major innate immune cells primarily responsive to SARS-CoV-2 infection.^20^ In this regard, an increased frequency of pre-neutrophils was similarly observed in four SARS-CoV-2^pos^ groups (Fig. 3a), implicating that the stimulated emergency myelopoiesis is an early and essential event post SARS-CoV-2 infection.^21^ Interestingly, the cDC subset was decreased in all four SARS-CoV-2^pos^ groups compared to that in the healthy donors while the pDC frequency was only decreased in the mild cases (Fig. 3b and Supplementary information, Fig. S4a). Meanwhile, similar to what was observed in lymphocytic subsets (Supplementary information, Fig. S2c, d), a higher CD44 level but a lower PD-1 level in the total DCs were observed in the asymptomatic group than those in the presymptomatic group (Supplementary information, Fig. S4b). These results showed sensitive alterations of DC subsets during the SSIS discrete from those observed in the acute phase.^22^

On the other hand, we identified four monocytic subsets based on differential expression of a number of surface functional markers including CD16, CD14 and CD107a (Fig. 3c). In addition to denoting the M03 cluster as the intermediate monocytes (I-Mo) and the M04 as the non-classical monocytes (NC-Mo), CyTOF separated the classical monocytes (C-Mo) into the M01 and M02 subsets based on their differential CD107a level. Given that the CD107a expression was correlated with IL-1β expression on the monocytes and also a well-accepted activation marker for immune cells,^23^ the CD107a^hi^ C-Mo might overlap with a previously reported IL-1β-expressing and abnormally activated inflammatory C-Mo subset exhibiting MDSC-like activity in COVID-19 patients.^24^ Adding to the previous findings that loss of the NC-Mo correlated with high severity of COVID-19 in acute phases,^21,25^ our observations showed that the total monocytic frequency was significantly decreased in the presymptomatic and moderate COVID-19 groups but not in the asymptomatic and mild COVID-19 groups compared to the healthy group (Fig. 3d). Specifically, the frequencies of CD107a^lo^ C-Mo, I-Mo and NC-Mo were all decreased in the presymptomatic group and mild/moderate groups as compared to the normal controls, while only the frequencies of I-Mo and NC-Mo were reduced in the asymptomatic group (Fig. 3e). Nevertheless, the frequencies of M02–M04 subsets were still significantly higher in the asymptomatic group than in the presymptomatic group, and an elevated ratio of the overactivated CD107a^hi^ C-Mo versus CD107a^lo^ C-Mo well separated the three COVID-19 groups from the normal controls and asymptomatic group (Fig. 3e). In this regard, the CD107a^hi^ C-Mo frequency selectively increased in the mild/moderate groups (Fig. 3e), and the pseudotime analysis indicated that the generation of the CD107a^hi^ C-Mo processed in a reverse way from the differentiation process of the CD107a^lo^ C-Mo through I-Mo to NC-Mo (Fig. 3f and Supplementary information, Fig. S4c). Moreover, the reduction of CD16 in the NC-Mo, indicative of NC-Mo differentiation retardation from the C-Mo through IM-Mo,^26^ was selectively seen in the presymptomatic group (Supplementary information, Fig. S4d). Taken together, these results indicated that an overactivation of C-Mo, accompanied with its retarded differentiation towards I-Mo and NC-Mo subsets, was more closely associated with the presymptomatic fate than the asymptomatic fate. Paradoxically, according to CD44 and PD-1 expression, the monocytic subsets were more activated in the asymptomatic group than in the presymptomatic group (Fig. 3g, h). Besides, the intracellular levels of IFN-γ, IL-4, IL-6 and IL-10 in monocytic cells and DCs were similarly reduced in four SARS-CoV-2^pos^ groups compared to the normal controls (Supplementary information, Fig. S4b). These observations indicated that the reduction of M02–M04 subsets in the presymptomatic group was accompanied with an aberrant activation pattern and compromised functionality.

In order to evaluate the relative contribution of the subset variations of CD4^+^ T cells, CD8^+^ T cells and monocytes to distinguishing the different groups of participants, a hierarchical clustering was performed through compositional similarity. Compared with CD4^+^ and CD8^+^ T cells across and within all the samples, the monocytic cluster composition showed the highest variability (Supplementary information, Fig. S4e), which was confirmed by Kullback-Leibler divergence analysis (Supplementary information, Fig. S4f). Accordingly, through Spearman correlation analyses of the frequencies of all immune clusters in all samples, we found that a strong positive association existed among three monocytic subsets of CD107a^lo^ C-Mo, I-Mo and NC-Mo subsets. In addition, positive correlation was also observed between CD8^+^ T_naïve_ cluster (T04) and MAIT (T14) as well as between CD107a^hi^ C-Mo and NKT (T15), while strong negative correlation was detected between NKT (T15) subset with CD107a^lo^ C-Mo, I-Mo, NC-Mo, or CD8^+^ T_naïve_ cluster (T04) (Fig. 3i, j and Supplementary information, Fig. S4g). These results indicated a potentially nodal role of abnormal monocytic activation and differentiation retardation in constructing a particular immune cell compositional alteration pattern that might foretell the preymptomatic cases from the asymptomatic cases at the SSIS.

### Proinflammatory monocytic overactivation associates with immunosuppression of the presymptomatic cases

**Fig. 4:**
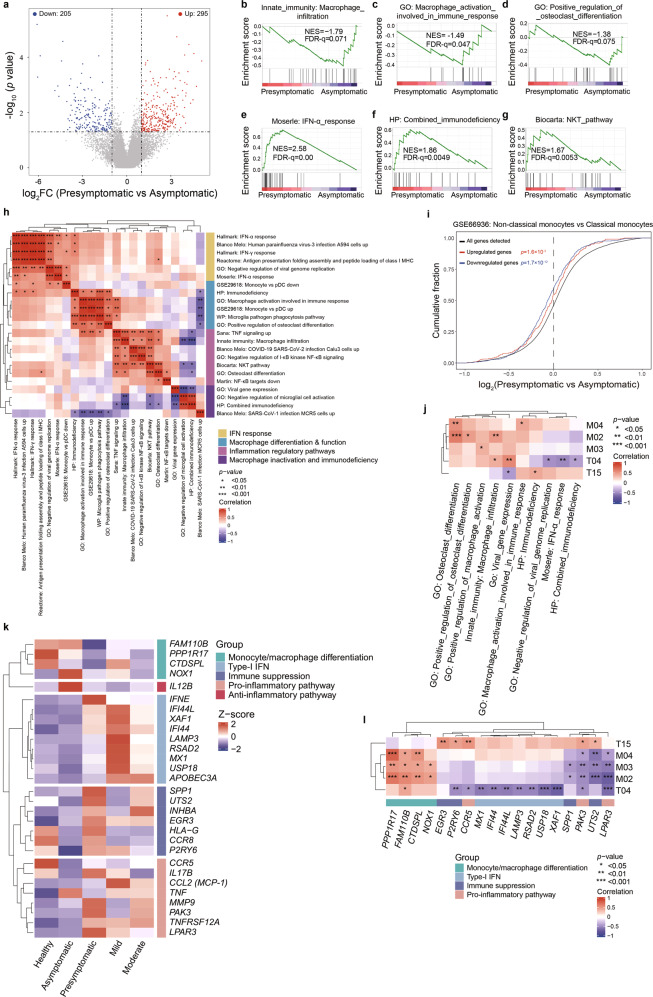
A few crucial PBMC-related signaling pathways differentially function in the asymptomatic and presymptomatic cases. **a** Volcano plot showing the genes differentially expressed (fold change ≥ 2, *P* < 0.05) in PBMCs between the presymptomatic versus asymptomatic cases. **b**–**g** GSEA of the expression profile of the presymptomatic or asymptomatic cases using macrophage infiltration-associated signature (**b**), macrophage activation-involved immune response-associated signature (**c**), osteoclast differentiation-associated signature (**d**), IFN-α response-associated signature (**e**), combined immunodeficiency-associated signature (**f**) and NKT pathway-associated signature (**g**). **h** Unsupervised hierarchical clustering of the Spearman correlation matrix of the gene sets differentially expressed between the asymptomatic and presymptomatic cases among SSIS groups. Gene set signature scores were calculated from mRNA expression as predefined linear combinations (weighted averages) of biologically relevant genes. **i** Empirical cumulative distribution function for the change in expression (log_2_ values) of all genes (black line) expressed in PBMCs of the presymptomatic subjects (change relative to that in PBMCs of asymptomatic subjects) and for subsets of genes upregulated (red line) or downregulated (blue line) in non-classical monocytes as compared with classical monocytes (GSE66936). **j** Heatmap showing Spearman correlations between the frequencies of indicated clusters and the enrichment of selected gene sets in the four SARS-CoV-2^pos^ groups. **k** Heatmap showing a number of genes differentially expressed between the presymptomatic and asymptomatic subjects. The functional categories of the genes are indicated. **l** Heatmap showing Spearman correlations between the frequencies of indicated clusters and the mRNA levels of selected genes in the four SARS-CoV-2^pos^ groups. The functional categories of the individual genes are indicated.

To comprehend the molecular mechanisms underlying immunological alterations post SARS-CoV-2 infection, we performed RNA-seq analysis of individual PBMC samples across the same cohorts. Akin to PCA analysis-based subset frequencies (Supplementary information, Fig. S2b), the mRNA profiling-based PCA showed that four groups of SARS-CoV-2^pos^ subjects or patients congregated together but separated from the healthy controls (Supplementary information, Fig. S5a). The highly related and top-ranked Kyoto Encyclopedia of Genes and Genomes (KEGG) pathways drawn from individually comparing SARS-CoV-2^pos^ groups with the normal group included those involving the viral protein, cytokine and cytokine receptor, and TNF signaling (Supplementary information, Fig. S5b–e). Interestingly, the osteoclast differentiation that probably reflected the NC-Mo differentiation from C-Mo was enriched only in two SSIS groups (Supplementary information, Fig. S5b–e), suggesting a possibility that the monocyte/macrophage differentiation blockade was specifically contributed to the reduced frequencies of I-Mo and NC-Mo subsets in the SSIS. To focus on comparing the asymptomatic with presymptomatic groups, our analysis identified 500 differentially expressed genes (Fig. 4a). Gene set enrichment analysis (GSEA) identified a number of top-ranked regulatory pathways that were enriched in the categories of the macrophage differentiation/activation/infiltration (Fig. 4b–d and Supplementary information, Fig. S5f–h), the type-I IFN signaling (Fig. 4e and Supplementary information, Fig. S5i), the immunodeficiency (Fig. 4f and Supplementary information, Fig. S5j), the lymphocyte exhaustion (Supplementary information, Fig. S5k),^27^ the NKT pathway (Fig. 4g), the pro-inflammatory pathways (Supplementary information, Fig. S5l, m), the viral replication inhibition (Supplementary information, Fig. S5n), the response to SARS-CoV-related viral infection (Supplementary information, Fig. S5o) and the antigen presentation (Supplementary information, Fig. S5p). Specifically, the infiltration, functional activation and differentiation potentials of several types of macrophages and the NF-κB activation were more active in the asymptomatic cases while the type-I IFN signaling, viral replication inhibition, antigen presentation, TNF and IFN-γ signaling, immunodeficiency, lymphocyte exhaustion, an NKT pathway, and response upon SARS-CoV-related viral infection were more positively associated with the presymptomatic cases (Fig. 4b–g and Supplementary information, Fig. S5f–p). Unsupervised hierarchical clustering of these gene signatures by Spearman analysis formed 4 distinctive modules concerning 1) IFN responses and restriction of viral replication, 2) macrophage differentiation and function, 3) mixed pathways consisting of TNF and NF-κB signaling pathways, macrophage differentiation/activation, NKT pathway and the responses to the SARS-CoV-2 infection, 4) macrophage inactivation and immunodeficiency (Fig. 4h). The frequent inclusion of macrophage differentiation, function and activation regulatory pathways into three out of four modules thus implicated nodal roles of the abnormal monocytic/macrophage activation, differentiation and functionality in constructing a likely pathogenic mechanistic hub that affected clinical courses of the two SSIS groups. The involvement of monocytic differentiation was further strengthened by the cumulative distribution analysis of public database (Fig. 4i and Supplementary information, Fig. S5q, r), which showed that the differential gene expression profiles of PBMCs in the presymptomatic versus the asymptomatic subjects were significantly associated with the downregulated expression profiles of the NC-Mo as compared to the C-Mo.

Then we explored how these regulatory pathways connect with key immune cell compositional alterations through inferring individual inter-correlations. IFN activation was reported to be associated with alterations in monocytes, megakaryocytes and erythroid cells as well as T cells in severe cases,^28–30^ while IFN signaling activation, along with immunodeficiency and viral replication inhibition, was specifically associated with the decreased CD62L^hi^ CD8^+^ T_naïve_ cell (T04) frequency in our cohorts (Fig. 4j). As expected, an immunodeficiency signature was positively associated with the frequency of CD4^+^ NKT cells with immunosuppressive potential, whereas a couple of macrophage differentiation, functional activation and infiltration-fueling pathways were positively associated with the frequencies of CD107a^lo^ C-Mo, NC-Mo and I-Mo subsets (Fig. 4j). To explore the potential molecular regulatory mechanisms, we then extracted and pooled the recurring genes behind these gene sets, which could be roughly grouped into five functional modules (Fig. 4k). As expected, the expression of *FAM110B*, *PPP1R17*, *CTDSPL* and *NOX1* that denotes the level of functional monocytic/macrophage differentiation and antiviral function was downregulated in the presymptomatic group compared with the asymptomatic group (Fig. 4k and Supplementary information, Fig. S5s, t).^31,32^ In contrast, type-I IFN-stimulated genes such as *XAF1*, *IFI44*, *IFI44L*, *LAMP3*, *RSAD2*, *MX1* and *USP18* as well as the immune suppression genes including *SPP1*, *UTS2*, *EGR3* and *P2RY6* were upregulated in the presymptomatic group (Fig. 4k).^33–39^ Of note, the pro-inflammatory pathway genes such as *CCR5*, *PAK3* and *LPAR3* that were implicated in the overactivation of MDSC-like CD107a^hi^ C-Mo were enriched in the presymptomatic group while the anti-inflammatory pathway gene *IL12B* was more abundant in the asymptomatic group (Fig. 4k),^40–42^ indicating a plausible causal link between the proinflammatory activation and enabled immunosuppression. Consistently, the monocytic differentiation genes were positively correlated with the frequencies of CD107a^lo^ C-Mo, I-Mo and NC-Mo, the type-I IFN-stimulated genes were negatively correlated with the CD8^+^ T_naïve_ cluster frequency, and the immune suppression and pro-inflammatory pathway genes were positive correlated with the CD4^+^ NKT subset frequency but negatively correlated with the CD8^+^ T_naïve_ cluster frequency (Fig. 4l). Interestingly, *PAK3* showed significant association with all 5 clusters (Fig. 4l), implicating that it may serve as a potential candidate for the future development of early therapeutic intervention at SSIS. Moreover, in consist with the aforementioned findings (Supplementary information, Fig. S2c–f), the immune exhausted marker *PD-1* and its upstream regulator *NFATC2*,^43^ as well as *TIGIT* and *NKG2A*, were also reduced in the asymptomatic group compared to normal subjects and/or the presymptomatic group (Supplementary information, Fig. S5u).

### Reduced STC1 plasma level is associated with the monocytic and lymphocytic abnormalities in the presympotomatic group

**Fig. 5:**
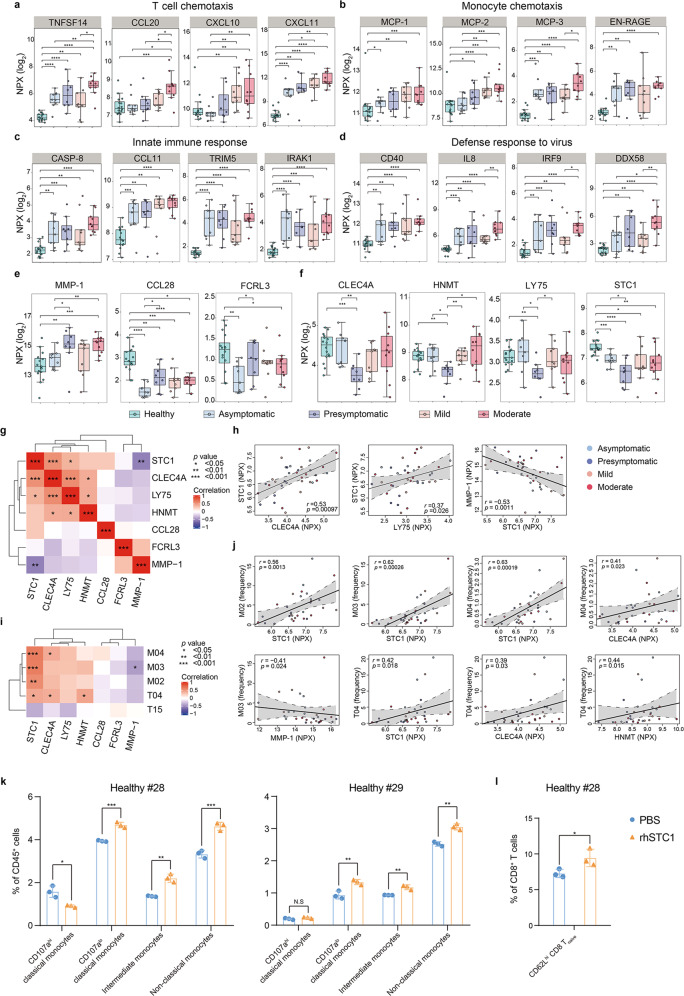
Characterization of the plasma inflammatory factors differentially presented in two SSIS groups. **a**–**d** Boxplots depicting the plasma levels of different types of inflammatory factors among the healthy controls, asymptomatic and presymptomatic subjects, mild and moderate COVID-19 patients. Unpaired Wilcoxon test was employed. **e**, **f** Boxplots of cytokines and other inflammatory factors reaching statistical significance in presymptomatic subjects compared with asymptomatic subjects. Unpaired Wilcoxon test was employed. **g** Heatmap showing Spearman correlations of the expression of indicated factors in the four SARS-CoV-2^pos^ groups. **h** Scatterplots showing relationships between the expression of selected inflammatory factors. **i** Heatmap showing Spearman correlations between frequencies of the indicated clusters and the expression of the selected inflammatory factors in the four SARS-CoV-2^pos^ groups. **j** Scatterplots showing relationships between the frequencies of the indicated clusters and the expression of the selected factors. **k**, **l** Flow cytometric analyses of the frequencies of four monocytic subsets within CD45^+^ cells (**k**) or CD62L^hi^ CD8^+^ T_naïve_ cells within CD8^+^ T cells (**l**) using BMMCs from healthy controls cultured with Serum-free Hematopoietic Cell Medium with or without rhSTC1 for 3 days. Unpaired Student’s *t-*test was employed. *P < 0.05, **P < 0.01, ***P < 0.001, ****P < 0.0001.

We also performed high-dimensional profiling of 180 different plasma proteins using the Olink platform to systemically characterize the secreted inflammatory factors in the SARS-CoV-2^pos^ subjects. Overall, obvious alterations in numerous inflammatory factors were observed in the SSIS as in the acute phase (Supplementary information, Fig. S6a–d). Interestingly, upregulation of inflammatory factors was highly overlapped in all four SARS-CoV-2^pos^ groups compared to the heathy group (25 out of 46) while those alterations occurring specifically to a sole SARS-CoV-2^pos^ group were much fewer (0–4 per situation) (Supplementary information, Fig. S6e). Accordantly, PCA analysis-based Olink profiling only separated the healthy controls, and to a weaker extent also separated the moderate group, from the rest (Supplementary information, Fig. S6f). In line with the aforementioned broad alterations involved in both lymphocytes and myeloid cells, we noticed that a number of the T cell or NK cell chemotactic factors including TNFSF14, CCL20, CXCL10 and CXCL11, and critical mediators of monocyte differentiation and activity such as MCP-1/CCL2, MCP-2, MCP-3 and EN-RAGE^44^ were elevated at least in three SARS-CoV-2^pos^ groups compared to the normal controls (Fig. 5a, b). Of them, elevations of TNFSF14, CXCL11 and all four monocyte regulatory factors became evident as early as at the SSIS, whereas the elevations of CCL20, CXCL10 and MCP-1 tended to be exacerbated in the acute stages. As expected, alterations of a portfolio of antiviral innate immune response-related factors such as CASP-8, CCL11, TRIM5, IRAK1, CD40, IL8, IRF9 and DDX58 were detectable in the SSIS and/or the acute phase of the COVID-19 (Fig. 5c, d), probably reflecting the activation of antiviral responses.

Interestingly, seven inflammatory factors including four monocyte-related ones (MMP-1, CLEC4A, LY75 and STC1) were identified to be differentially presented between the asymptomatic and presymptomatic groups (Fig. 5e, f).^45–48^ Of a particular interest in verifying the monocytic overactivation of the presymptomatic cases (Fig. 3e, f), the level of MMP-1 indicative of monocytic activation in the presymptomatic group but not the asymptomatic group was significantly higher than that in the healthy controls (Fig. 5e),^49^ whereas a more severely reduced level of STC1, a critical monocytic activation-restriction factor by counteracting MCP-1-exerted activity on NC-Mo activation and migration and also a negative regulator of MMP-1,^48,50^ was selectively seen in the presymptomatic group (Fig. 5f). In accordance, the STC1 level was positively associated with the levels of CLEC4A and LY75, two potential monocytic functional markers, but negatively correlated with the MMP-1 level. All these factors together form a monocytic differentiation-regulatory module (Fig. 5g, h).^51,52^ These observations suggested a likely activity of STC1 in preventing the COVID-19 severity-related monocytic overactivation and differentiation blockade. Likewise, the significantly positive correlations of the STC1 level with the frequencies of all three monocytic subsets (CD107a^lo^ C-Mo, I-Mo and NC-Mo) further supported this notion (Fig. 5i, j).^26^ Finally, a positive correlation existing between STC1 level and the CD8^+^ T_naïve_ cell frequency likely implicated a plausible causal connection between monocytic overactivation and the T cell lymphopenia in the SSIS.^53^ In line with these assumptions, recombinant human STC1 (rhSTC1) administration significantly increased the frequencies of CD107a^lo^ C-Mo, I-Mo and NC-Mo as well as CD62L^hi^ CD8^+^ T_naïve_ cells in the bone marrow mononuclear cell (BMMC) culture (Fig. 5k, l and Supplementary information, Fig. S7a, b), supporting the notion that STC1 drove the monocytic differentiation while maintaining the CD62L^hi^ CD8^+^ T_naïve_ frequency.

### Two immunological modules discriminate the asymptomatic subjects from the presymptomatic cases

**Fig. 6:**
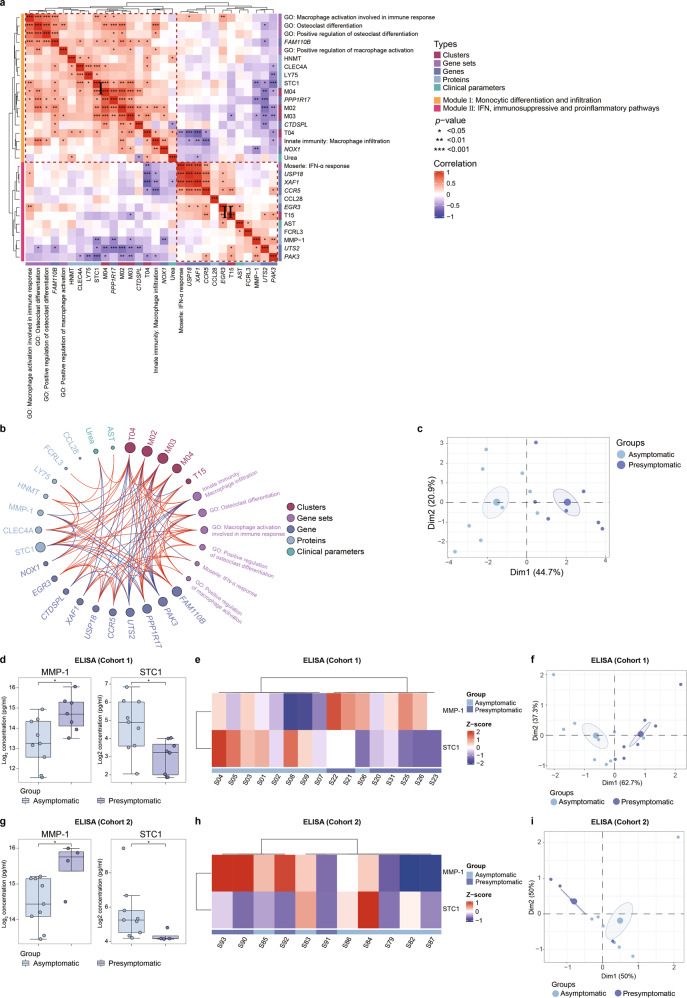
An immune signature composite well separates two SSIS groups. **a** Unsupervised hierarchical clustering of the Spearman correlation matrix of 5 immune cell clusters, 6 gene sets, 10 genes, 7 cytokines and 2 clinical parameters for the four SARS-CoV-2^pos^ groups. **b** Circos plot visualizing the Spearman correlation matrix of 5 immune cell clusters, 6 gene sets, 10 genes, 7 cytokines and 2 clinical parameters for the four SARS-CoV-2^pos^ groups. Each line represents a significant correlation with *P* < 0.05. Lines indicate positive (red) or negative (red) correlations. Size of nodes indicates degree centrality, with larger nodes representing higher degree. **c** PCA of the 4 immune cell clusters (T04, M02, M03 and M04), 2 gene sets (innate immunity: macrophage infiltration; GO: osteoclast differentiation), 5 genes (*FAM110B*, *PAK3*, *PPP1R17*, *UTS2* and *CCR5*), and 1 cytokine (STC1) for the asymptomatic and presymptomatic subjects. Each dot represents a subject, colored by disease status. **d** Concentration of plasma MMP-1 and STC1 measured by ELISA in the asymptomatic and presymptomatic subjects of the Cohort 1. **e** Heatmap depicting the expression of MMP-1 and STC1 measured by ELISA in the asymptomatic and presymptomatic subjects of the Cohort 1. **f** PCA of the expression of MMP-1 and STC1 measured by ELISA in the asymptomatic and presymptomatic subjects of the Cohort 1. Each dot represents a subject, colored by disease status. **g** Concentration of plasma MMP-1 and STC1 measured by ELISA in the asymptomatic and presymptomatic subjects of the Cohort 2. **h** Heatmap depicting the expression of MMP-1 and STC1 measured by ELISA in the asymptomatic and presymptomatic subjects of the Cohort 2. **i** PCA of the expression of MMP-1 and STC1 measured by ELISA in the asymptomatic and presymptomatic subjects of the Cohort 2. Each dot represents a subject, colored by disease status. Significance in **d**, **g** was determined by unpaired Wilcoxon test. *P < 0.05.

Probably due to interference from a great number of noisy SSIS-related parameters, the PCA based on whole datasets of CyTOF, RNA-seq and Olink failed to separate the asymptomatic subjects from the presymptomatic cases (Supplementary information, Figs. S2b, S5a, S6f). Nevertheless, a 5-parameter panel based on measuring the frequencies of C-Mo, I-Mo, NC-Mo, CD8^+^ T_naïve_ and CD4^+^ NKT exhibited increased capacity of discriminating between the asymptomatic group and presymptomatic group at the SSIS (Supplementary information, Fig. S8a, b). To improve this, we then added six gene sets (five sets for macrophage differentiation, activation and infiltration as well as one for type-I IFN response), ten genes (*FAM110B*, *PPP1R17*, *CTDSPL* and *NOX1* for denoting monocytic differentiation and functionality; *USP18* and *XAF1* for type-I IFN signaling; *CCR5* and *PAK3* for pro-inflammatory pathway; *EGR3* and *UTS2* for immuno-repressive activity), all seven plasma proteins that were differentially presented in two SSIS groups (Fig. 5e, f), two serum parameters (urea and AST) into the five cell cluster panel mentioned above. Interestingly, unsupervised clustering of these composite factors (*n* = 30) based on their intercorrelations formed two large modules (Fig. 6a), with the bigger one enriched with monocytic/macrophage differentiation and infiltration markers as well as their regulatory pathways/factors, and the minor one consisting of activation markers of type-I IFN signaling pathways (visibly more closely related), immunosuppressive pathways and proinflammatory pathways. Thus, two relatively independent pathogenic or protective immune processes functioned at the SSIS; the presymptomatic cases were positively associated with a mixed activation of type-I IFN signaling, proinflammatory pathway and immunosuppressive pathway, whereas the asymptomatic cases retained normal differentiation and functionality of monocytes and CD8^+^ T_naïve_ cells. As expected, PCA analysis based on two distinct dimensions of these 30-factor panel bettered the separation of the asymptomatic subjects from the presymptomatic cases in our cohorts (Supplementary information, Fig. S8c, d).

Last but not the least, the Circos plot visualization assigned four immune cell subsets (CD8^+^ T_naïve_ cells, CD107a^lo^ C-Mo, I-Mo and NC-Mo), monocytic/macrophage differentiation and infiltration, five genes (*FAM110B*, *PPP1R17, PAK3*, *UTS2* and *CCR5*), and STC1 to more central positions within a putatively highly interactive immunoregulatory network that potentially governed the fate choice of SARS-CoV-2^pos^ subjects at the SSIS (Fig. 6b). Notably, the unsupervised clustering and PCA analysis based on these 12 nodal factors well differentiated the asymptomatic subjects from the presymptomatic cases (Fig. 6c and Supplementary information, Fig. S8e, f). These results also further implicated that a few of them such as STC1, *PAK3* and *CCR5* might act as potential candidate targets for developing prophylactic interventions of the presymptomatic cases.

ELISA-based protein detection in blood plasma with fewer markers is desired for clinical diagnosis in the real world. By analyzing the protein expression of Olink proteomics, we found that two key monocyte-related markers STC1 and MMP-1 virtually were able to well discriminate the asymptomatic group from presymptomatic group at the SSIS (Fig. 5e, f and Supplementary information, Fig. S9a, b), which was verified by ELISA (Fig. 6d–f). To validate this, more blood plasma samples of asymptomatic and presymptomatic subjects at SSIS in another independent cohort (the Cohort 2) were tested (Supplementary information, Fig. S9c, d and Table S9). Notably, the ELISA assay on the plasma levels of STC1 and MMP-1 well differentiated the asymptomatic subjects from presymptomatic subjects at the SSIS in the Cohort 2, as well as in the combined cohorts (Fig. 6g–i and Supplementary information, Fig. S9e, f).

## Discussion

Over last year, enormous numbers of reports focused on the acute phase and convalescent phase of COVID-19 patients have unveiled several featured immune responses in association with disease severity and recovery, shedding light on developing targeted immune adjustments to help manage those severe COVID-19 patients.^6^ Nevertheless, the phenomenon that a major part of SARS-CoV-2^pos^ cases virtually experienced a completely asymptomatic course has remained as an unsolved mystery. Our identification of an immune signature composite capable of discriminating the asymptomatic cases from the presymptomatic cases will facilitate the practice of a more effective quarantine and early intervention strategy that will block the COVID-19 transmission at a more efficient and economical way.

Given that the reported median incubation time from SARS-CoV-2 exposure to symptom onset is usually 4–5 days (although the incubation phase may be up to 2 weeks), which is too narrow to allow the full activation of adaptive immunity,^10^ our findings that obvious alterations to the circulating lymphocytes had already taken place in both the asymptomatic and presymptomatic subjects as in the two acute phase groups sound quite unexpected. The major reason could be that all enrolled participants in our study were young adults without comorbidities who tended to have longer silent infection time. The large-scale disturbance of lymphocytic composition and functionality at the SSIS might also come from 1) an antigen recognition-independent stimulation by proinflammatory cytokines generated by activated monocytes and macrophages and 2) a T/B cell memory reaction to previously exposed certain cross-reaction antigens derived from other types of coronaviruses.

A featured monocytic/macrophage overactivation-centered scenario has been proposed, in which the monocytic/macrophage overactivation would result in a severe cytokine release syndrome, to be companied with reduced NC-Mo frequency, activated but functionally compromised T lymphocytes, and a likely vicious disease progression.^8^ In support of this, a few previously well-documented inhibitors of monocytic/macrophage overactivation exhibited therapeutic benefits.^54,55^ Relevantly, our cohort study based on systemic profiling of PBMCs and plasma with combined assays at cellular, RNA and protein levels revealed a particular pattern of monocytic abnormalities and SSIS-related underlying mechanisms. The abnormal overactivation of C-Mo (as indicated by the ratio of CD107a^hi^ C-Mo versus CD107a^lo^ C-Mo) decreased the frequency of normal CD107a^lo^ C-Mo upon SARS-CoV-2 infection, and RNA-seq analysis highlighted that the monocytic/macrophage differentiation retardation impeded the generation and proper functional activation of I-Mo and NC-Mo that were probably required for exerting protective effects to ensure an asymptomatic fate. As for underlying mechanisms, unlike those prevailing in the acute phases such as type-I IFN, IL-1 and IL-6 signaling pathways,^28^ we identified a critical role of the deeply reduced serum STC1 in fueling the pathogenic monocytic abnormality of the presymptomatic cases. The tight association of monocytic abnormalities with the characteristic loss of CD8^+^ T_naïve_ cells in the presymptomatic cases suggested a causal relationship between them. Besides, an enhanced intracellular type-I IFN signaling probably underlay the CD8^+^ T_naïve_ cell reduction, although evidence of increased type-I IFN generation was missing as shown by many previous COVID-19 studies.^30^

Particularly, we found that a potentially immunosuppressive CD4^+^ NKT cellular subset was selectively increased in the presymptomatic cases. The frequency of this NKT subset was positively and tightly associated with an immune module involving the activation of proinflammatory pathways rather than with the other monocytic or T lymphocytic module just described above. Its specific association with the certain proinflammatory factors such as *PAK3* and *CCR5* provides a potential intervention avenue to test its contribution to the establishment of presymptomatic fate. On the other hand, it is interesting to investigate in the future whether ectopic upregulation of CD44 but downregulation of PD-1 selectively in certain types of immune cells of the asymptomatic cases represents a kind of positive response of host to balance the SARS-CoV-2 infection-emanated interference for the proper activation and functionality of immune cells.

## Materials and methods

### Patient information

All SARS-CoV-2^pos^ subjects and COVID-19 patients who were confirmed with SARS-CoV-2 infection assay according to the Centers for Disease Control (CDC) in Shanghai were admitted to the Clinic of Shanghai Public Health Clinical Center from March 2020 to November 2020. Healthy donors with RT-PCR test negative for SARS-CoV-2 and no virus-specific serum IgM and IgG were recruited from Ruijin Hospital, affiliated to Shanghai Jiao-Tong University School of Medicine. All the volunteers mentioned above were between 18 to 40 years old, had no comorbidities (such as hypertension, cardiovascular disease, diabetes, hyperlipidemia, malignancy, cerebrovascular disease, chronic obstructive pulmonary disease, asthma, chronic renal disease, chronic liver disease and HIV/AIDS), and received no treatment of antibiotics, antiviral drugs and glucocorticoids over two weeks before enrollment and peripheral blood sampling. General information, including demography, comorbidities, typical and atypical symptoms of COVID-19 and medication history, was provided by the recruited individuals themselves, and clinical data of SARS-CoV-2^pos^ subjects or patients were collected from the electronic medical record. Peripheral blood was collected from all the participants in three days upon recruitment. Routine blood test, immunological test and measurement of the serum SARS-CoV-2-specific IgM and IgG of healthy donors were performed. Besides, blood biochemistry, coagulation and infection biomarkers were assessed in asymptomatic and presymptomatic subjects as well as symptomatic COVID-19 patients in three days after being admitted to hospital. Chest computed tomography (CT) were assessed repeatedly in asymptomatic subjects and symptomatic COVID-19 patients before discharge.

105 individuals who agreed to participate and fulfilled the inclusion/exclusion criteria, were enrolled in the study. Participants were assigned to the study groups as follows. 1) Healthy donors who were negative for SARS-CoV-2 and virus-specific IgM and IgG as indicated by RT-PCR test (27 healthy donors). 2) The seemingly healthy subjects who were tested as SARS-CoV-2^pos^ but manifested no symptoms over two recent weeks (31 patients). These subjects were quarantined, treated with hydroxychloroquine, azvudine, antibiotics, Chinese medicinal herb, Vitamin C and thymosin and monitored for > 14 days, and the quarantine ended when at least two consecutive nasopharyngeal swabs showed negative results of SARS-CoV-2 detection. A group of them manifesting no symptoms and signs through whole observation period was denoted as the asymptomatic cases (*n* = 19), whereas a group of subjects diagnosed with pneumonia by chest CT during the observation period were classified as pre-symptomatic cases (*n* = 12). 3) Patients with mild COVID-19 (23 patients), defined by positive results in nasopharyngeal swab RT-PCR test for SARS-CoV-2, with symptoms such as fever, cough, myalgia, and fatigue but without obvious abnormalities on chest CT images. 4) Patients with moderate COVID-19 (24 patients), defined by positive results in nasopharyngeal swab RT-PCR test for SARS-CoV-2, with symptoms such as fever, cough, myalgia and fatigue and pneumonia diagnosed by chest CT but fulfilling the exclusion criteria of severe type of COVID-19, including respiratory rate ≤ 30 per min, oxygen saturation on room air at rest ≥ 93% and partial pressure of oxygen in arterial blood /fraction of inspired oxygen ≥ 300 mmHg.

In the Cohort 2, 11 asymptomatic individuals and 4 presymptomatic cases fulfilled the inclusion/exclusion criteria of our study were included. They were SARS-CoV-2^pos^ subjects who were confirmed with SARS-CoV-2 infection assay according to the CDC in Shanghai and admitted at the Clinic of Shanghai Public Health Clinical Center from November 2020 to May 2021. All the volunteers were between 18 to 40 years old, had no comorbidities (such as hypertension, cardiovascular disease, diabetes, hyperlipidemia, malignancy, cerebrovascular disease, chronic obstructive pulmonary disease, asthma, chronic renal disease, chronic liver disease and HIV/AIDS), and received no treatment of antibiotics, antiviral drugs and glucocorticoids over two weeks before enrollment and peripheral blood sampling. General information, including demography, comorbidities, typical and atypical symptoms of COVID-19 and medication history, was provided by the recruited individuals themselves, and clinical data of SARS-CoV-2^pos^ subjects were collected from the electronic medical record. Peripheral blood was collected from all the participants in three days upon recruitment. Routine blood test, immunological test and measurement of the serum SARS-CoV-2-specific IgM and IgG of healthy donors were performed. Chest CT were assessed repeatedly in asymptomatic and presymptomatic subjects before discharge.

### Specimen preparation

All peripheral blood samples were collected into heparin vacutainer tubes (Becton Dickinson). Tubes were spun (10 min, 3000 rpm, room temperature (RT)), and plasma was collected and stored at –80 °C. PBMCs were isolated by density gradient centrifugation using lymphocyte separation medium (TBDscience), washed twice with Ca/Mg-free PBS (BasalMedia) and cryopreserved in medium containing 90% fetal calf serum (FCS) (Gibco), 10% dimethyl sulfoxide (Sigma-Aldrich) until using.

PBMCs from 16 healthy controls, 9 asymptomatic subjects, 7 presymptomatic subjects, 8 mild COVID-19 patients and 8 moderate COVID-19 patients were processed for CyTOF. Plasma from 16 healthy controls, 9 asymptomatic subjects, 8 presymptomatic subjects, 9 mild COVID-19 patients and 10 moderate COVID-19 patients were processed for Olink. Total RNA extracted from PBMCs of 15 healthy controls, 9 asymptomatic subjects, 7 presymptomatic subjects, 7 mild COVID-19 patients and 9 moderate COVID-19 patients were used for RNA-seq. 28 SARS-CoV-2^pos^ subjects could be simultaneously analyzed by CyTOF, Olink and RNA-seq (Supplementary information, Table S1).

### Flow cytometry

CD3^+^ T, CD4^+^ T, CD8^+^ T, CD19^+^ B, and CD16^+^ CD56^+^ NK cells were stained using BD Multitest 6-color TBNK reagent in Trucount tubes. All anti-human antibodies were purchased from BD Biosciences: CD45-APC-Cy™7 (#557833), CD3-APC (#555342), CD4-FITC (#566802), CD8-Percp-cy5.5 (#565310), CD14-AF488 (#562689), CD16-PE (#561313), CD107a-APC (#560664), CD45RA-BV605 (#562886), CD62L-PE (#555544), CD197-BV421 (#566743). Total cells were Fc-blocked and stained with indicated combinations of antibodies for 30 min on ice, then washed three times and resuspended in 1% FBS/PBS. The flow cytometric data were collected on a BD Calibur flow cytometer and analyzed using FlowJo software or Summit software.

### Anti-SARS-CoV-2 antibody detection

Serum samples of patients were tested for IgM/IgG antibodies against COVID-19 using the colloidal gold immunochromatography antibody detection kit (Vazyme Biotech) according to the manufacturer’s instructions.

### Antibodies and antibody labeling

The total 44 metal-conjugated antibodies and corresponding provider and clone are listed in Supplementary information, Table S7. Metal-labeled antibodies were purchased or prepared using the Maxpar MCP9 Antibody Labeling Kit (Fluidigm) and Maxpar X8 Antibody Labeling Kit (Fluidigm) according to the manufacturer’s instructions. The concentration of each antibody was determined using a Nanodrop (Thermo Scientific) after conjugation. Metal-conjugated antibodies were diluted in HRP-Protector peroxidase stabilizer (Candor Bioscience) and Antibody Stabilizer PBS (Candor Bioscience), respectively, to a concentration of 0.5 mg/mL for long-term storage at 4 °C. Antibody labeling was performed by National Research Center for Translational Medicine at Shanghai or PTL-Tech lnc. (Hangzhou, China). Proper staining concentrations for antibodies were determined by titration.

### Antibody staining for PBMCs

To ensure homogeneous staining, ~2 × 10^6^ to 3 × 10^6^ PBMCs were used each time for each patient. PBMCs were stained with Cisplatin (Fluidigm) (0.1 μL, 2 min, RT) for live/dead, washed with Cell Staining Buffer (CSB) (Fluidigm), and spun down (300× *g*, 5 min, RT). Then, PBMCs were incubated with Human TruStain FcX (Biolegend) for 10 min at RT. After incubation, PBMCs were stained with 50 μL surface receptor staining mix (30 min, RT), and washed twice with CSB (300× *g*, 5 min, RT). Next, PBMCs were incubated with Fix I buffer (Fluidigm) for 15 min at RT and washed twice with Perm-S buffer (Fluidigm) (800× *g*, 5 min, RT). PBMCs were stained with 50 μL intracellular mix (30 min, RT) and washed twice with CSB (800× *g*, 5 min, RT). PBMCs were then fixed in 1 mL 1.6% paraformaldehyde (PFA). Samples were fixed and permeabilized by incubating 1 mL Fix and Perm buffer (Fluidigm) with 1 μL nucleic acid Ir-Intercalator (Fluidigm) overnight at 4 °C.

### CyTOF data acquisition

Before acquisition, PBMCs were washed twice by CSB and then resuspended at a concentration of 1.1 × 10^6^ cells/mL in Cell Acquisition Solution (Fluidigm) containing 10% of EQ Four Element Calibration Beads (Fluidigm). The PBMCs were acquired on the Helios CyTOF Mass Cytometer (Fluidigm) equipped with a SuperSampler fluidics system (Victorian Airships), and data were collected as .fcs files. CyTOF analyses were performed by National Research Center for Translational Medicine at Shanghai.

### CyTOF data analysis

After acquisition, data were concatenated using the.fcs concatenation tool from Cytobank, and manually gated to retain live, singlet, valid immune cells. CytoNorm was performed in two steps following the instruction provided in the R library CytoNorm to normalize the data.^56^ For downstream analysis,.fcs files were loaded into R. Signal intensities for each channel were arcsinh transformed with a cofactor of 5 (x_transf = asinh(x/5)). In order to visualize the high-dimensional data, t-SNE^57^ and FlowSOM^58^ algorithms were performed on all samples. 10,000 cell events in each individual sample have been pooled and included in t-SNE analysis, a perplexity of 30, and a theta of 0.5. The R t-SNE package for Barnes-Hut implementation of t-SNE was used. Data were displayed using the ggplot2 R package.

To visualize expression analyses on t-SNE maps, the expression (*y*) was normalized between 0 and 1. *y* = (value – minimum)/(maximum – minimum); minimum = 3% quantile, maximum = 97% quantile. *y* is 0 for all *y* smaller than 0; *y* is 1 for all *y* bigger than 1. The data from all samples were divided by this *y* value leading to signal intensities ranging between 0 and 1 for each channel. Clustering analysis was performed using FlowSOM run on all samples simultaneously. To identify the main cell subsets in the datasets, FlowSOM was run with the parameter *k* = 35, defining the number of clusters. Heatmaps were displayed in R using complexheatmap, and the expression was normalized between 0 and 1 as mentioned above.

For Monocle 2 analysis,^59^ the dataset of marker intensities in individual cells was arcsinh transformed with a cofactor of 5 (x_transf = asinh(x/5)), combined, and analyzed using the ‘monocle’ R package available on BioConductor. A cellDataSet was created with ‘uninormal’ as the family function parameter. Typically, monocle defaults to a negative binomial. The data were already normalized, thus the ‘uninormal’ parameter was used. Dimensionality reduction was done to a maximum of 2 components utilizing the ‘DDRTree’ method, after which cells were ordered to obtain a pseudotime trajectory. This trajectory was then plotted, with a smooth line fitted along the trajectory. Cells were also colored according to clusters.

### Plasma protein profiling using Olink multiplex panel

Protein arrays were performed on 52 plasma samples which were heat-inactivated at 56 °C for 15 min to inactivate the SARS-CoV-2. Concentrations of 180 proteins were assessed by proximity extension assay (Olink Bioscience, Sweden) using the Inflammation panel and Immune Response panel (Olink proteomics: www.olink.com). The assay used two matched oligonucleotide-labeled antibodies allowing for pair-wise binding to target proteins. When antibody pairs bind specific antigens, corresponding oligonucleotides form an amplicon allowing for quantification of protein expression by microfluidic qPCR using Fluidigm Biomark HD sysytem. Data were presented as normalized protein expression values, Olink Proteomics’ arbitrary unit on a log_2_ scale. Olink analyses were performed by National Research Center for Translational Medicine at Shanghai.

### RNA-seq

Approximately 1 million live PBMCs after thaw were solubilized in 1 mL of Trizol. The samples were frozen at −80 °C until RNA isolation. Total RNA was extracted from PBMCs using Trizol reagent (Invitrogen) according to the manufacturer’s instructions. RNA-seq transcriptome library was prepared following KAPA Stranded mRNA-Seq Kit (KAPA Biosystems, Wilmington, MA, USA) using 1 μg of total RNA. Shortly, isolation of mRNA was performed using the KAPA PolyA mRNA capture beads and the mRNA was then used for library preparation with the KAPA Stranded RNA-Seq Library Preparation Kit for Illumina® platforms (KAPA Biosystems, Wilmington, MA, USA). The library was then subjected to Illumina sequencing with paired-end 2 × 150 as the sequencing mode, paired-end RNA-seq library was sequenced with the Illumina Nova-seq (2 × 150 bp read length). For bioinformatics analyses, raw sequence reads were initially processed using FastQC (Babraham Institute, Cambridge, UK) for quality control, and then adapter sequences and poor-quality reads were removed using Cutadapt (v1.9.1) and Trimmomatic (v0.35). Quality-filtered reads were then mapped to human genome (hg38) using HISAT2 software, and only the uniquely mapped reads were kept. Read counts were calculated using StringTie. Differentially expressed genes were identified using R package edgeR.

### GSEA and KEGG

Gene annotation file was retrieved from Ensembl genome browser 96 database (http://www.ensembl.org/index.html). The functional enrichment analysis of KEGG was performed with ClusterProfiler. GSEA was performed using the GSEA software v4.0.3 (Broad Institute, Cambridge, USA).^60^

### PCA

PCA was performed using the R packages FactoMineR and factoextra.^61^ Data were centered and scaled before principal components (PCs) were computed. The individual data points were plotted using the fviz_pca_ind function.

### Data sources for in silico analyses

For the cumulative distribution function analysis, mRNA expression data of the differentially expressed genes between the isolated non-classical monocytes versus classical monocytes from PBMCs of the healthy controls were retrieved from a previous report^62^ (GEO accession codes: GSE66936, GSE106757 or GSE106840). The mRNA expression data for *FAM110B* or *PPP1R17* in 29 immune cell types and total PBMCs were analyzed by the online tools resided in The Human Blood Atlas, retrieved from GEO (GSE107011) dataset.^63^

### Cell culture

The human BMMCs from healthy donors were isolated by density gradient centrifugation using lymphocyte separation medium (TBDscience). To analyze the effects of STC1 on monocytic differentiation, BMMCs were cultured with X-VIVO™ 15 Serum-free Hematopoietic Cell Medium (LONZA; 04-418Q) containing 50 ng/mL hSCF (R&D Systems; 255-SC-050) or/and 200 ng/mL rhSTC1 (R&D Systems; 9400-SO-050) in 5% CO_2_ and humidified atmosphere at 37 °C for 3 days. To analyze the effect of STC1 on CD62L^hi^ CD8^+^ T_naïve_ cells, BMMCs were cultured with X-VIVO™ 15 Serum-free Hematopoietic Cell Medium (LONZA; 04-418Q) containing 200 ng/mL hIL-2 (R&D Systems; 202-IL-050) or/and 200 ng/mL rhSTC1 (R&D Systems; 9400-SO-050) in 5% CO_2_ and humidified atmosphere at 37 °C for 3 days.

### ELISA

Cytokine concentrations were measured using specific ELISA kits, including those for human STC1 (Abcam; ab213829) and human MMP-1 (RayBiotech; ELH-MMP-1) according to the manufacturer’s instructions.

### Statistical analysis

Considering the heterogeneity of clinical and flow cytometric data, unpaired Wilcoxon tests were performed throughout this study unless otherwise specified. Corrplot R package was used for Spearman’s rank correlation analysis in order to identify phenotypically similar clusters. Heatmaps were created to visualize variable values using R function *pheatmap* or *complexheatmap*. Significance was indicated by *P < 0.05, **P < 0.01, ***P < 0.001, ****P < 0.0001.

## Supplementary information

Supplementary information, Figure S1

Supplementary information, Figure S2

Supplementary information, Figure S3

Supplementary information, Figure S4

Supplementary information, Figure S5

Supplementary information, Figure S6

Supplementary information, Figure S7

Supplementary information, Figure S8

Supplementary information, Figure S9

Supplementary information, Table S1

Supplementary information, Table S2

Supplementary information, Table S3

Supplementary information, Table S4

Supplementary information, Table S5

Supplementary information, Table S6

Supplementary information, Table S7

Supplementary information, Table S8

Supplementary information, Table S9

## Data Availability

The raw sequence data of RNA-seq reported in this paper have been deposited in the Genome Sequence Archive of the BIG Data Center at the Beijing Institute of Genomics, Chinese Academy of Science, under the accession number HRA000786 (accessible at http://bigd.big.ac.cn/gsa-human). All other relevant data are available from the corresponding author on request.
