# Supplementary material for: Distinct immune signatures discriminate between asymptomatic and presymptomatic SARS-CoV-2pos subjects
Source: Cell Res. 2021 Sep 24;31(11):1148–62. doi: 10.1038/s41422-021-00562-1 (PMC8461439; doi:10.1038/s41422-021-00562-1)
Supplement: Supplementary file 1 — Supplementary information, Figure S1 [file 41422_2021_562_MOESM1_ESM.pdf]

Supplementary information, Figure S1

a

	SARS-CoV-2 infections	
	Asymptomatic subjects (n=19)	Presymptomatic subjects (n=12)
The duration of viral shedding, days	7.00 (4.00-13.00)	12.00 (6.00-16.25)
Days from admission to appearance of pneumonia imaging findings (the incubation period), days	N/A	6.00 (4.25-9.00)
Hospitalization days	13.00 (11.00-16.00)	16.00 (13.00-23.00)
SARS-CoV-2 IgM or IgG in serum, no./total no. (%)		
Positive	12/19 (63.2)	10/12 (83.3)
Negative	7/19 (36.8)	2/12 (16.7)

Data are shown as median (IQR) unless otherwise noted.

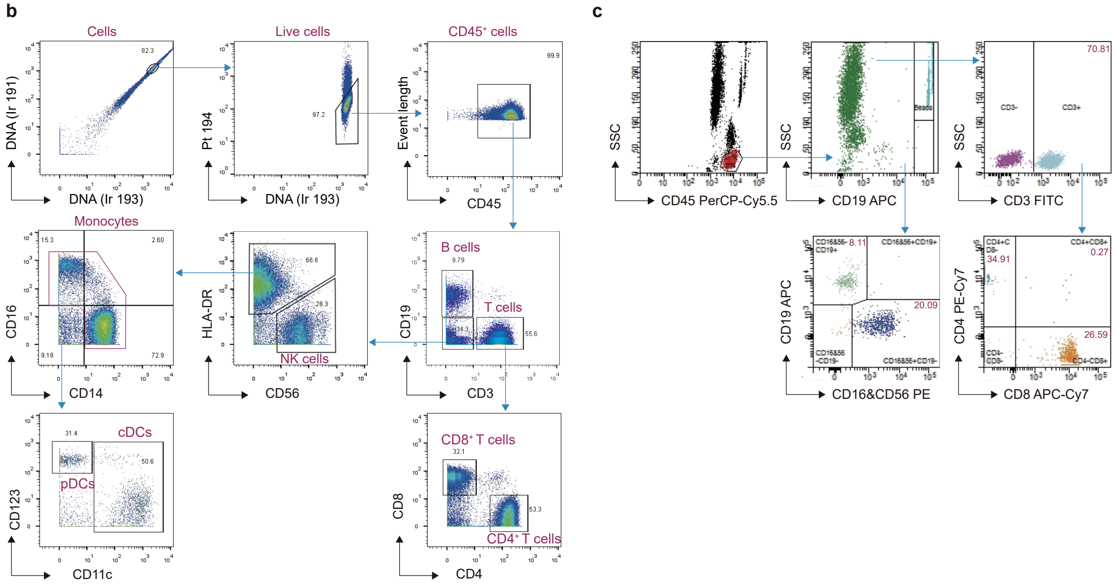

Supplementary information, Figure S1. Key clinical parameters of SARS-CoV-2^{pos} subjects at the silent infection stage and the immune cell type-gating strategy of CyTOF or flow cytometry.

a The clinical characteristics of the 19 asymptomatic and 12 presymptomatic subjects enrolled in our study, related to Figure**1b**. **b** PBMCs were isolated by density centrifugation and processed by CyTOF, and the gating strategy to identify live cells and the major immune cell types from PBMCs by CyTOF is shown. **c** Whole blood samples were subjected to red blood cell lysis and processed by flow cytometry, and the gating strategy to identify the major lymphocytic cell types by flow cytometry is shown.
