# Supplementary material for: Distinct immune signatures discriminate between asymptomatic and presymptomatic SARS-CoV-2pos subjects
Source: Cell Res. 2021 Sep 24;31(11):1148–62. doi: 10.1038/s41422-021-00562-1 (PMC8461439; doi:10.1038/s41422-021-00562-1)
Supplement: Supplementary file 2 — Supplementary information, Figure S2 [file 41422_2021_562_MOESM2_ESM.pdf]

Supplementary information, Figure S2

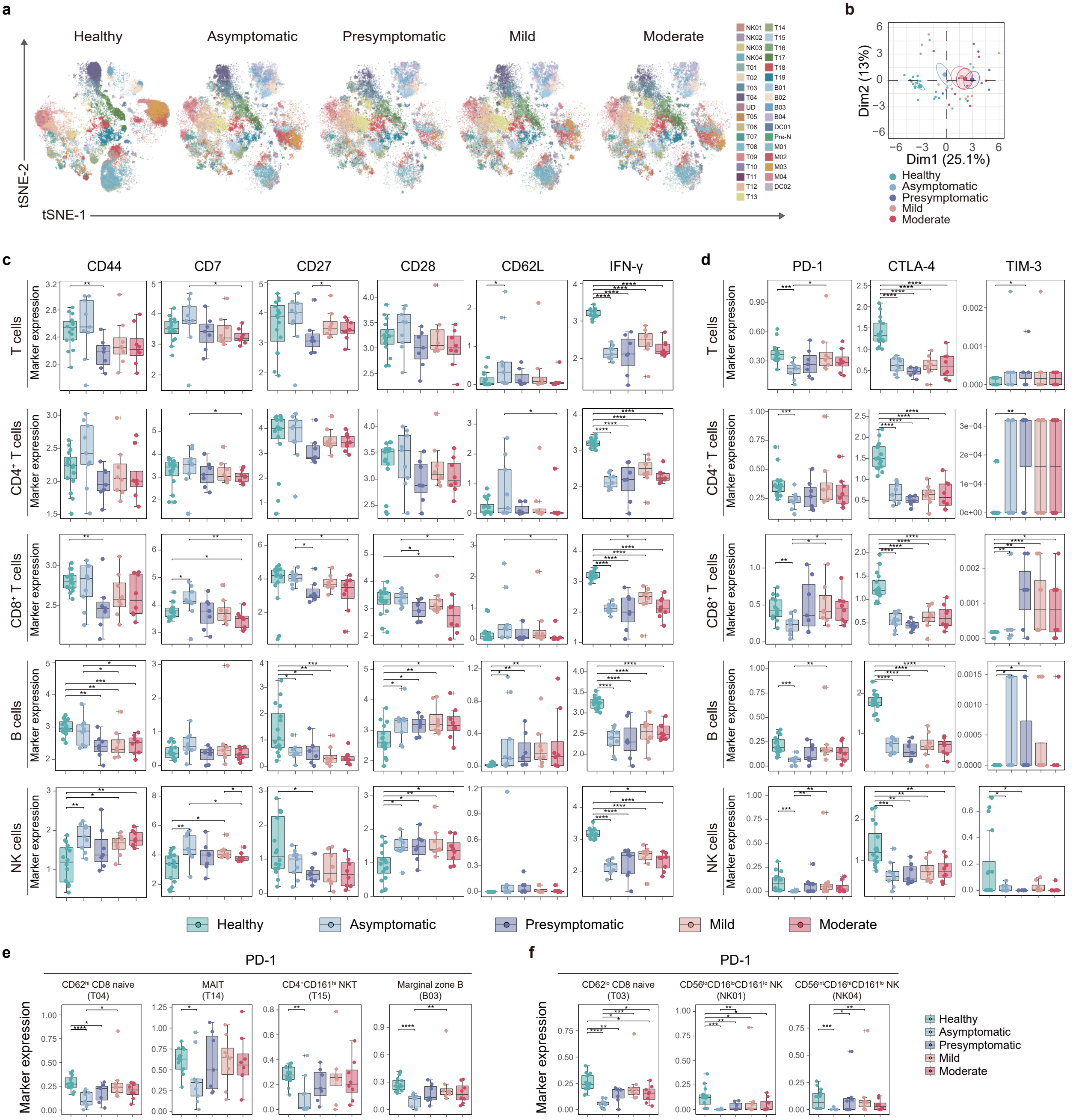

Supplementary information, Figure S2. CyTOF assay on the expression of functional markers in lymphocytes.

a t-SNE map displaying immune cells pooled from 10,000 cellular events in each individual sample. Cell identities are colored by PhenoGraph cluster. **b** Principal component analysis (PCA) of the participants based on the variation of 35 immune cell cluster frequencies across the groups. Each dot represents a participant, colored by disease status. **c, d** Boxplots showing the expression levels of indicated markers in the total T cells, $CD4^+$ T cells, $CD8^+$ T cells, B cells and NK cells across the groups. **e, f** Boxplots showing the expression level of PD-1 in the particular immune cell clusters across the groups. Significance was determined by unpaired Wilcoxon test. $^*p < 0.05$, $^{**}p < 0.01$, $^{***}p < 0.001$, $^{****}p < 0.0001$.
