# Supplementary material for: Distinct immune signatures discriminate between asymptomatic and presymptomatic SARS-CoV-2pos subjects
Source: Cell Res. 2021 Sep 24;31(11):1148–62. doi: 10.1038/s41422-021-00562-1 (PMC8461439; doi:10.1038/s41422-021-00562-1)
Supplement: Supplementary file 3 — Supplementary information, Figure S3 [file 41422_2021_562_MOESM3_ESM.pdf]

Supplementary information, Figure S3

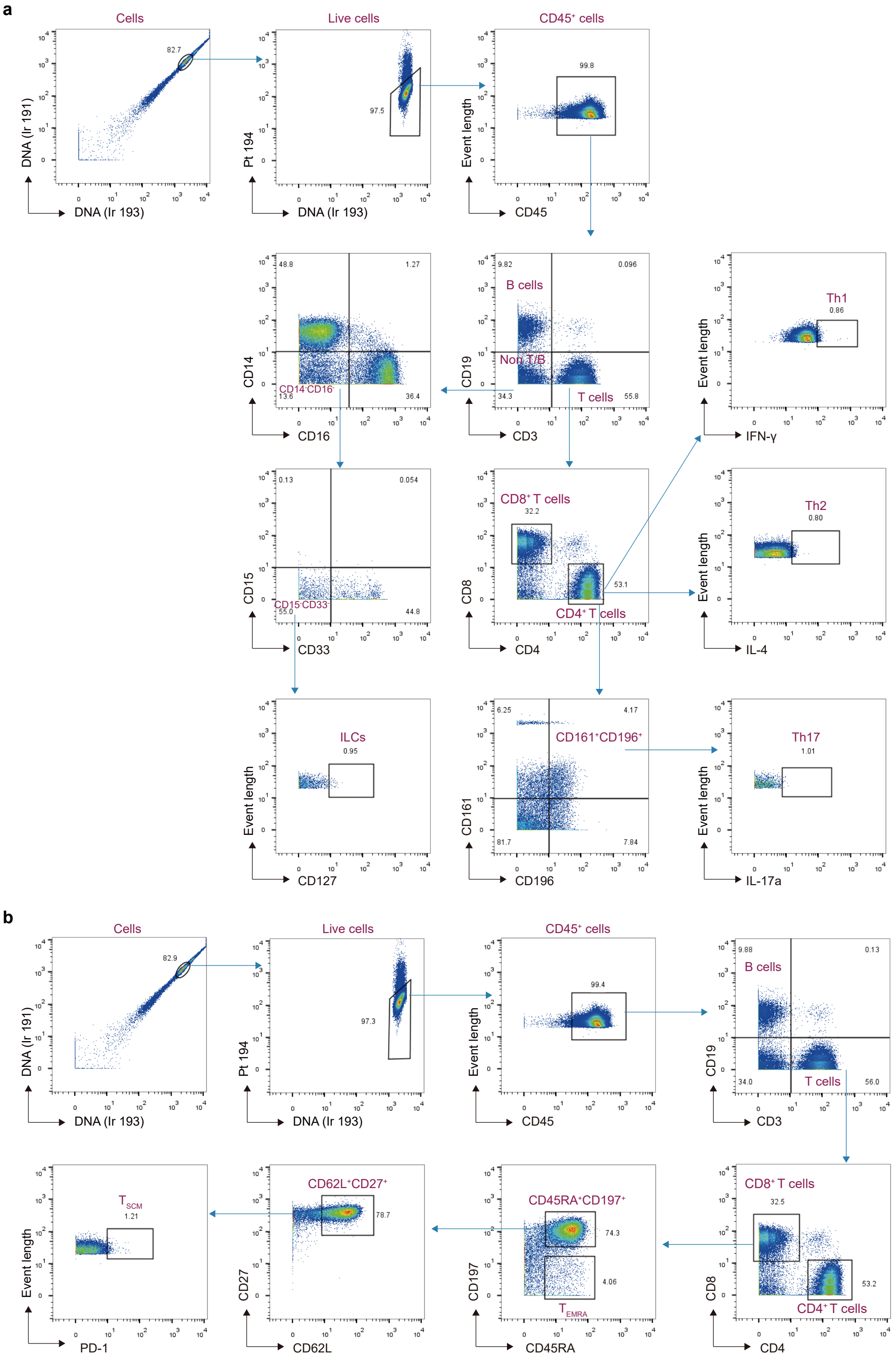

Supplementary information, Figure S3. Gating strategy to identify specific lymphocytic subsets by CyTOF.

a PBMCs were isolated by density centrifugation and processed by CyTOF, and the manual gating strategy to identify ILCs ($CD45^+CD3^-CD19^-CD14^-$ $CD16^-CD15^-CD33^-$ $CD127^+$), Th1 ($CD45^+CD3^+CD4^+IFN\text{-}\gamma^+$), Th2 ($CD45^+CD3^+CD4^+IL\text{-}4^+$) and Th17 ($CD45^+CD3^+CD4^+CD161^+CD196^+IL\text{-}17^+$) from PBMCs by CyTOF is shown. **b** PBMCs were isolated by density centrifugation and processed by CyTOF, and the manual gating strategy to identify $CD8^+$ T_{SCM} ($CD45^+CD3^+CD8^+CD45RA^+CD197^+CD62L^+CD27^+PD\text{-}1^+$) and $CD8^+$ T_{EMRA} ($CD45^+CD3^+CD8^+CD45RA^+CD197^-$) from PBMCs by CyTOF is shown.
