# Supplementary material for: Distinct immune signatures discriminate between asymptomatic and presymptomatic SARS-CoV-2pos subjects
Source: Cell Res. 2021 Sep 24;31(11):1148–62. doi: 10.1038/s41422-021-00562-1 (PMC8461439; doi:10.1038/s41422-021-00562-1)
Supplement: Supplementary file 4 — Supplementary information, Figure S4 [file 41422_2021_562_MOESM4_ESM.pdf]

Supplementary information, Figure S4

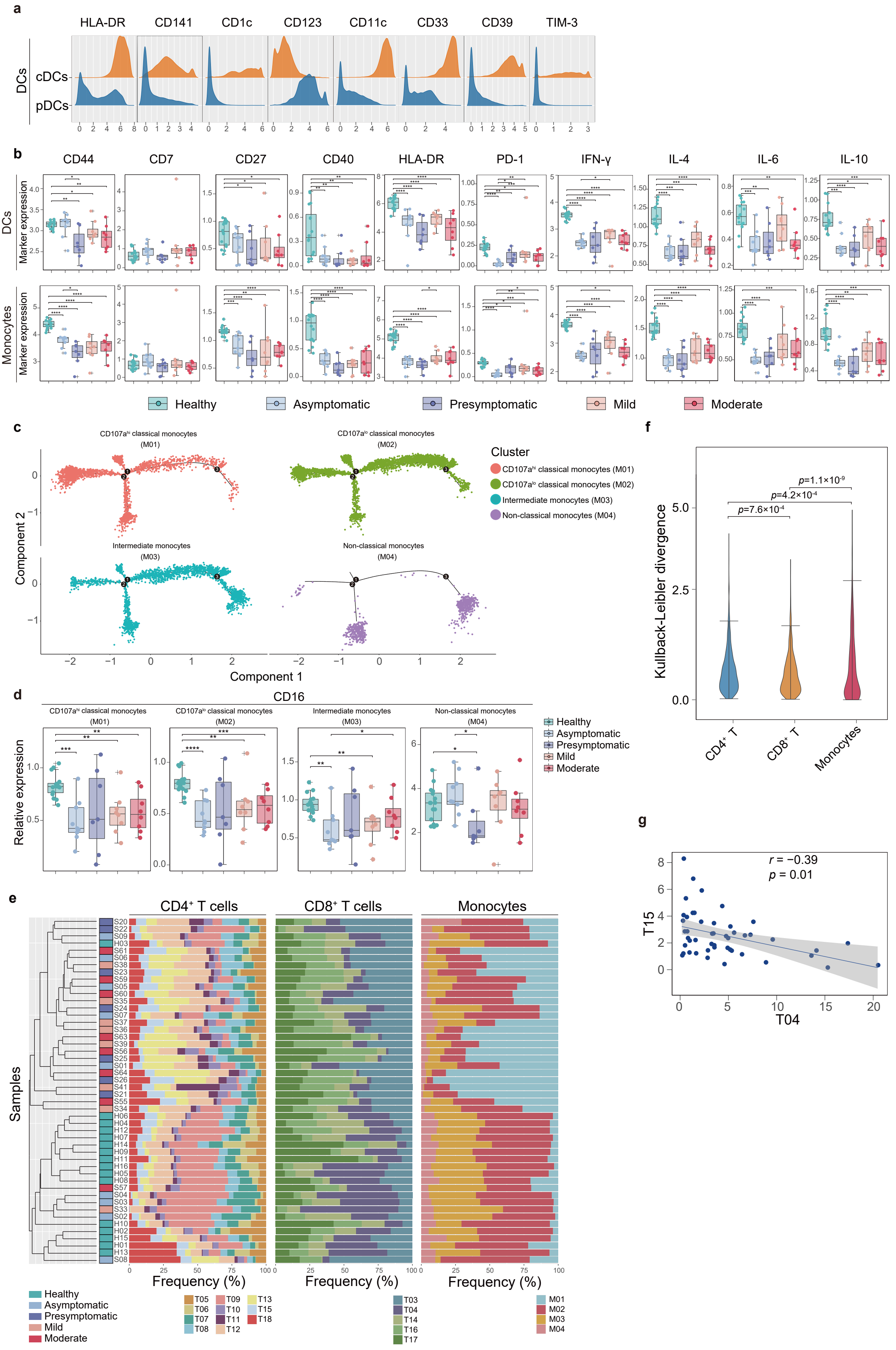

Supplementary information, Figure S4. The expression profile of functional marker in myeloid cells.

a Histograms depicting the expressions of indicated DC lineage markers and coinhibitory molecules in DC clusters. **b** Boxplots showing the expression of indicated markers in DCs and monocytes across the groups. **c** Monocle 2 trajectory analysis was performed on a combined dataset of each monocyte cluster across the groups, monocle plot displays monocytes color-coded by different monocytic clusters. **d** Boxplots showing the expression of CD16 in the monocytic subsets across the groups. **e** Clustering data hierarchically of $CD4^+$ and $CD8^+$ T cells and monocytes using Ward's methods. Sample types are indicated by color. **f** Violin plot showing the Kullback-Leibler divergence computed for each patient for $CD4^+$ and $CD8^+$ T cells and monocytes compartments. The Welsch *t* test was used to calculate differences between means, and the *p* value is shown for each relationship. **g** Scatterplot showing relationship between the frequencies of $CD8^+$ T_{naive} (T04) and $CD4^+$ NKT (T15) across all the participants. For significant correlation, linear model is shown as blue lines. **b**, **d** significance was determined by unpaired Wilcoxon test. *$p < 0.05$, **$p < 0.01$, ***$p < 0.001$, ****$p < 0.0001$.
