# Supplementary material for: Distinct immune signatures discriminate between asymptomatic and presymptomatic SARS-CoV-2pos subjects
Source: Cell Res. 2021 Sep 24;31(11):1148–62. doi: 10.1038/s41422-021-00562-1 (PMC8461439; doi:10.1038/s41422-021-00562-1)
Supplement: Supplementary file 5 — Supplementary information, Figure S5 [file 41422_2021_562_MOESM5_ESM.pdf]

Supplementary information, Figure S5

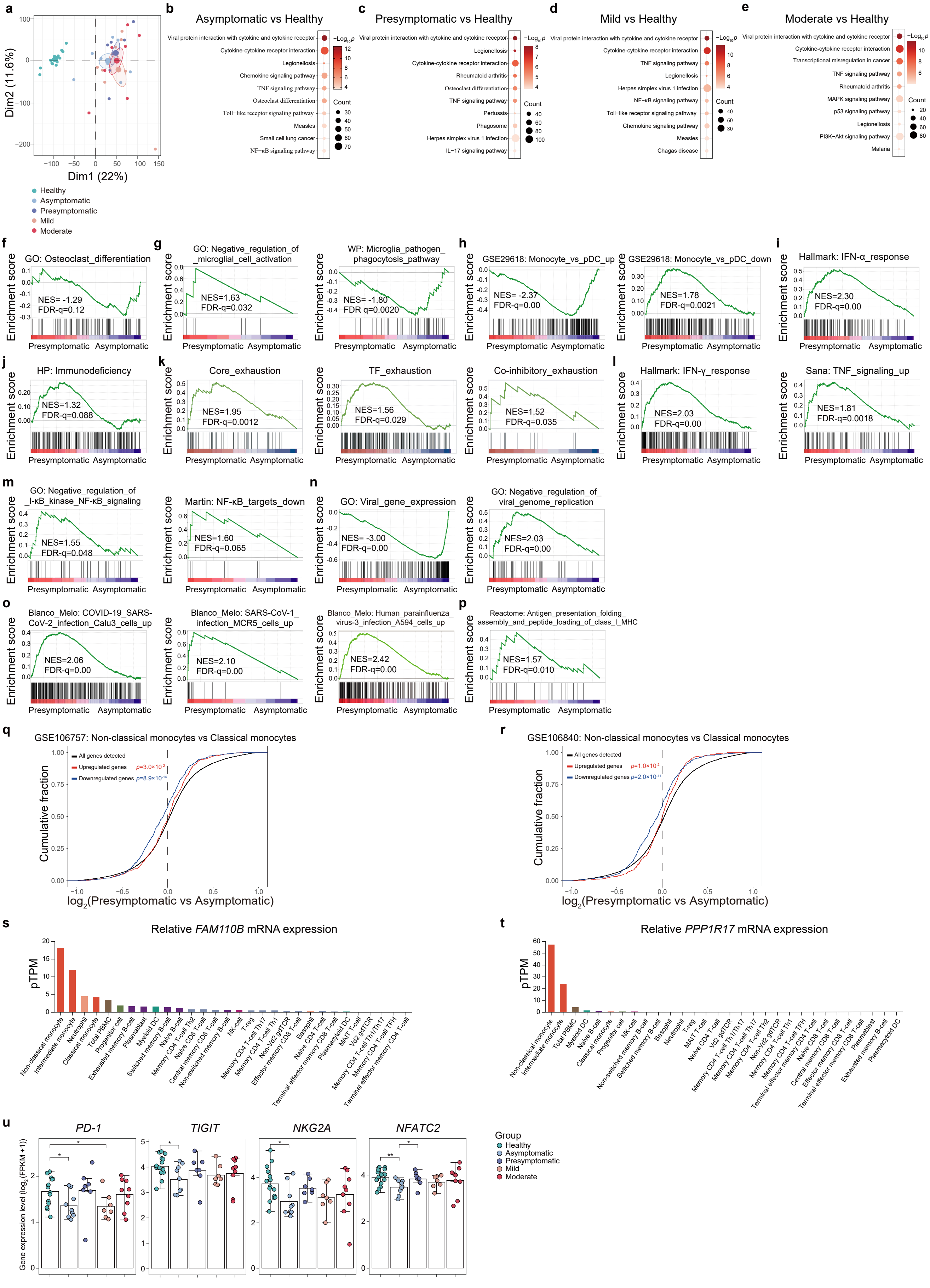

Supplementary information, Figure S5. PCA analysis and characterization of key signaling pathways potentially related to the COVID-19 disease severity.

a PCA analysis of RNA-seq datasets across the groups. Each dot represents a participant, colored by disease status. **b**-**e** The top 10 KEGG pathways of the differentially expressed genes (fold change ≥ 2, $p < 0.05$) between asymptomatic subjects and healthy controls (**b**), between presymptomatic subjects and healthy controls (**c**), between mild patients and healthy controls (**d**), and between moderate patients and healthy controls (**e**). The size of the dot indicates the number of the differentially expressed genes enriched in the pathway, and the color indicates the *p* value. **f**-**m** GSEA of the expressing profile of the presymptomatic or asymptomatic subjects using osteoclast differentiation-associated signature (**f**), microglial cell activation-negative associated signature (**g**, left panel), microglia pathogen phagocytosis pathway-associated signature (**g**, right panel), monocyte versus pDC upregulated (**h**, left panel) or downregulated signature (**h**, right panel), IFN-α response-associated signature (**i**), immunodeficiency-associated signature (**j**), core exhaustion-associated signature (**k**, left panel), TF exhaustion-associated signature (**k**, middle panel), co-inhibitory exhaustion-associated signature (**k**, right panel), IFN-γ response-associated signature (**l**, left panel), TNF signaling-associated upregulated signature (**l**, right panel), NF-κB signaling-negative associated signature (**m**, left panel), or NF-κB targets-associated downregulated signature (**m**, right panel), viral gene expression-associated signature (**n**, left panel), viral genome replication-negative associated signature (**n**, right panel), SARS-CoV-2 infection-associated upregulated signature (**o**, left panel), SARS-CoV-1 infection-associated upregulated signature (**o**, middle panel), respiratory parainfluenza virus-3 infection-associated upregulated signature (**o**, right panel), antigen presentation folding assembly and peptide loading of class I MHC-associated signature (**p**). **q**, **r** Empirical cumulative distribution function for the change in expression ($\log_2$ values) of all genes (black line) expressed in PBMCs of the presymptomatic subjects (change relative to that in PBMCs of asymptomatic subjects) and for subsets of genes upregulated (red line) or downregulated (blue line) in non-classical monocytes as compared with classical monocytes obtained from GSE106757 (**q**) or GSE106840 (**r**). **s**, **t** Relative *FAM110B* (**s**) or *PPP1R17* (**t**) mRNA expression

levels on 29 immune cell types and total PBMCs, the mRNA levels for *FAM110B* or *PPP1R17* were analyzed by the online tools resided in The Blood Atlas, raw data retrieved from GEO (GSE107011) dataset. pTPM, transcripts per million of total transcripts from protein-coding genes. **u** Relative gene expression levels of lymphocyte exhaustion-related genes across the groups. Significance was determined by unpaired Wilcoxon test. *$p < 0.05$, **$p < 0.01$.
