# Supplementary material for: Distinct immune signatures discriminate between asymptomatic and presymptomatic SARS-CoV-2pos subjects
Source: Cell Res. 2021 Sep 24;31(11):1148–62. doi: 10.1038/s41422-021-00562-1 (PMC8461439; doi:10.1038/s41422-021-00562-1)
Supplement: Supplementary file 6 — Supplementary information, Figure S6 [file 41422_2021_562_MOESM6_ESM.pdf]

Supplementary information, Figure S6

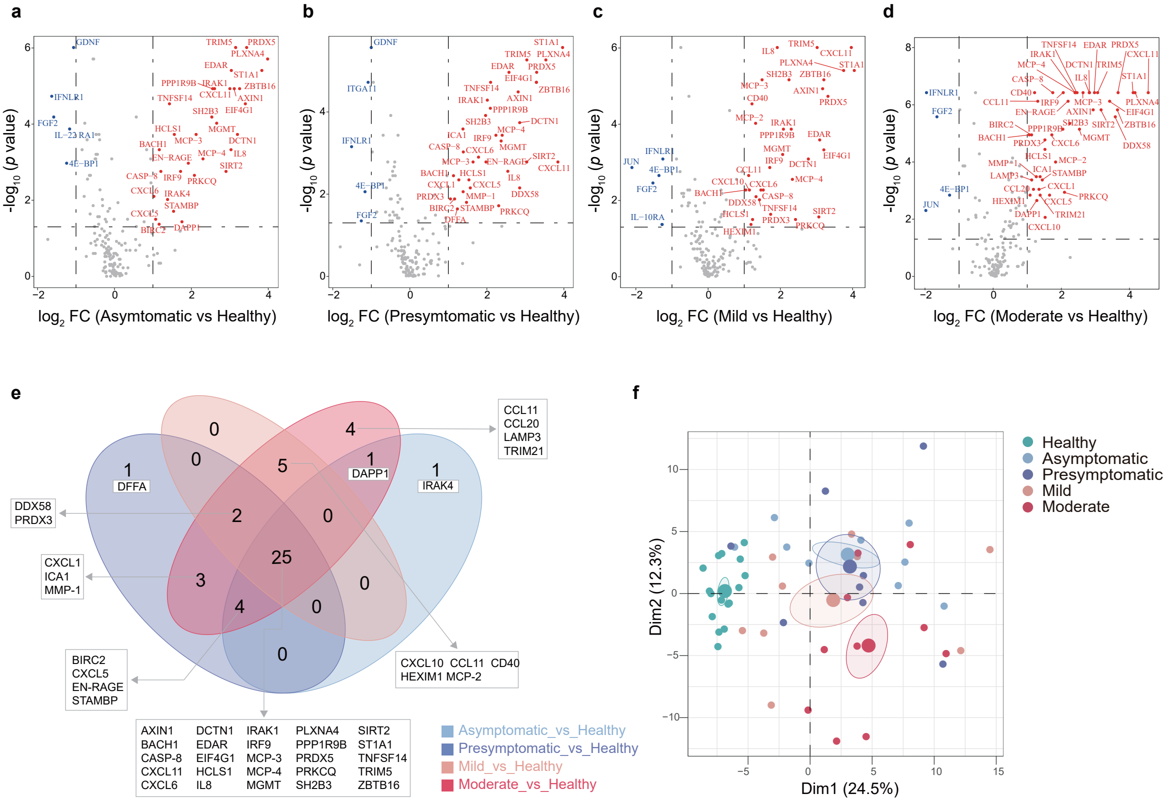

Supplementary information, Figure S6. Profiling of plasma inflammatory factor levels in association with disease status.

a-**d** Volcano plots showing plasma inflammatory factors differentially expressed (fold change ≥ 2, $p < 0.05$) between the asymptomatic subjects and healthy controls (**a**), between the presymptomatic subjects and healthy controls (**b**), between the mild patients and healthy controls (**c**), and between the moderate patients and healthy controls (**d**). **e** Venn diagram of the upregulated plasma cytokines differentially expressed (fold change ≥ 2, $p < 0.05$) between asymptomatic subjects and healthy controls, between presymptomatic subjects and healthy controls, between mild patients and healthy controls, or between moderate patients and healthy controls. **f** PCA analysis of plasma proteins across the groups. Each dot represents a participant, colored by disease status.
