# Supplementary material for: Distinct immune signatures discriminate between asymptomatic and presymptomatic SARS-CoV-2pos subjects
Source: Cell Res. 2021 Sep 24;31(11):1148–62. doi: 10.1038/s41422-021-00562-1 (PMC8461439; doi:10.1038/s41422-021-00562-1)
Supplement: Supplementary file 7 — Supplementary information, Figure S7 [file 41422_2021_562_MOESM7_ESM.pdf]

Supplementary information, Figure S7

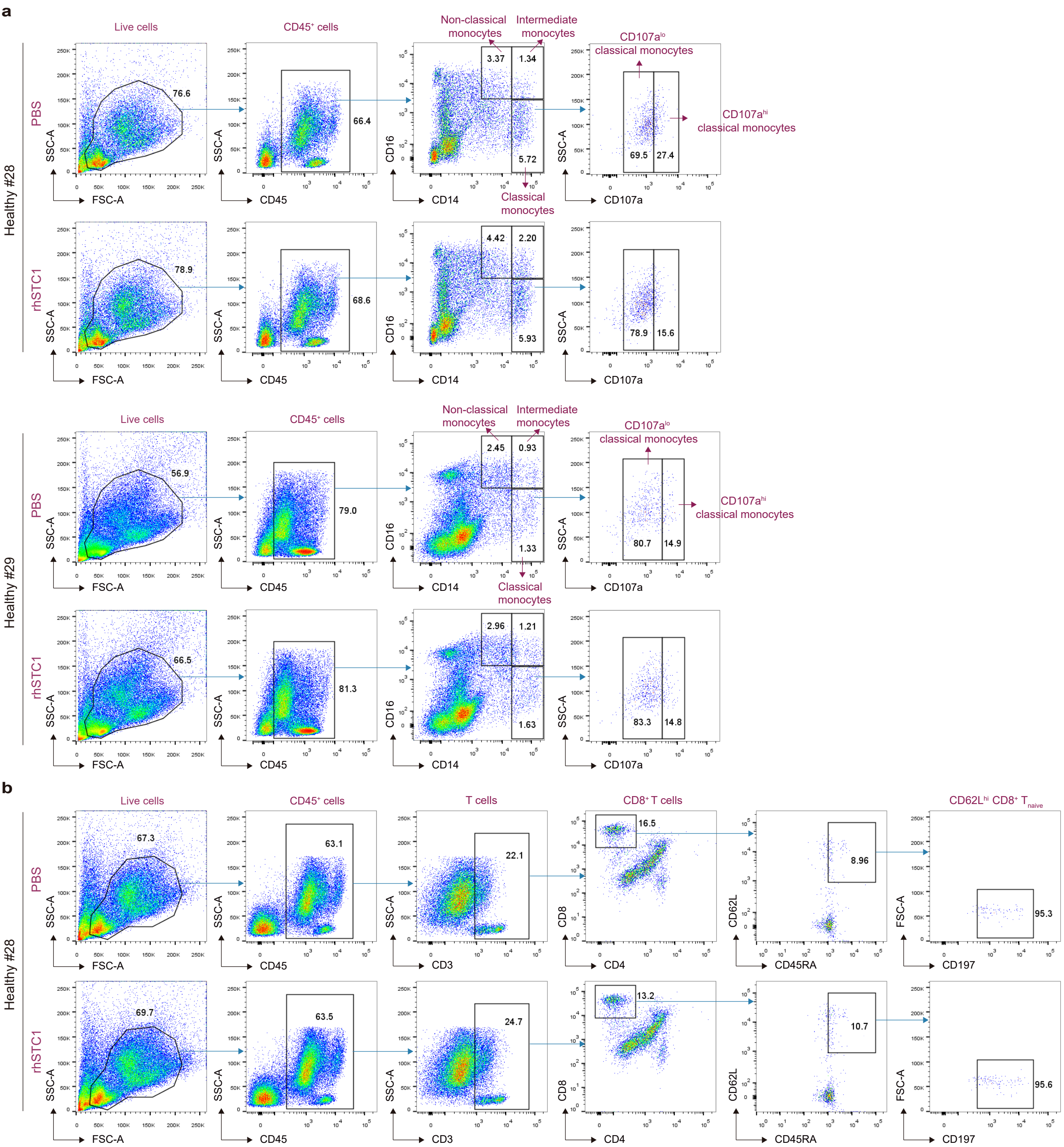

Supplementary information, Figure S7. The function of STC1 on monocytic and lymphocytic abnormalities.

a, b Analyses of four monocytic subsets (**a**) or CD62L^{hi} CD8^{+} T$_{naive}$ cells (**b**) in BMMCs from healthy controls cultured with Serum-free Hematopoietic Cell Medium with or without rhSTC1 using flow cytometric assay.
