# Supplementary material for: Distinct immune signatures discriminate between asymptomatic and presymptomatic SARS-CoV-2pos subjects
Source: Cell Res. 2021 Sep 24;31(11):1148–62. doi: 10.1038/s41422-021-00562-1 (PMC8461439; doi:10.1038/s41422-021-00562-1)
Supplement: Supplementary file 8 — Supplementary information, Figure S8 [file 41422_2021_562_MOESM8_ESM.pdf]

Supplementary information, Figure S8

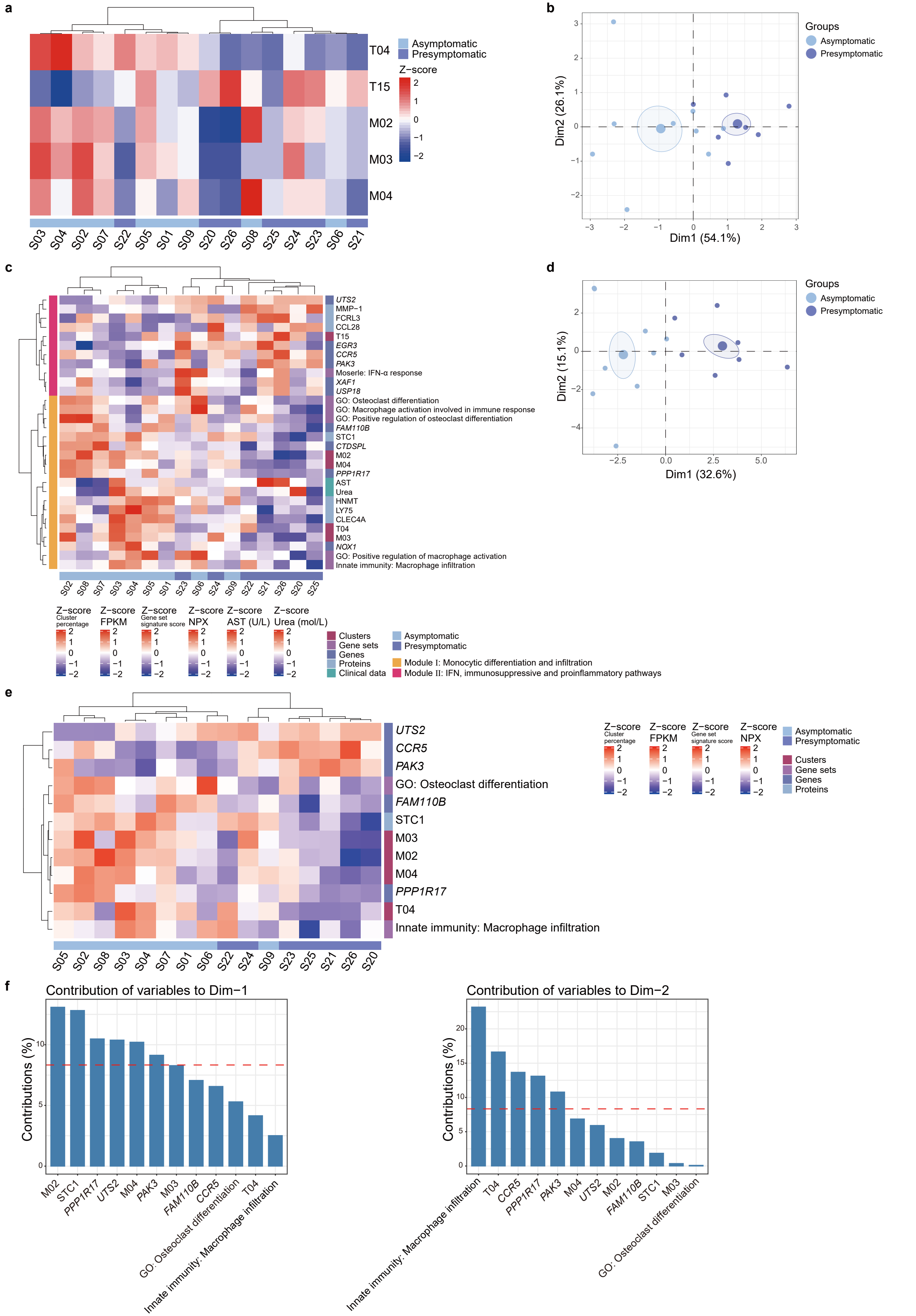

Supplementary information, Figure S8. The prediction power of the five immune cell clusters or 12 nodal factors at SSIS.

a Heatmap depicting the frequencies of 5 immune cell clusters in the asymptomatic and presymptomatic subjects. **b** PCA of the frequencies of 5 immune cell clusters (**a**) in the asymptomatic and presymptomatic subjects. Each dot represents a subject, colored by disease status. **c** Integrated unsupervised hierarchical clustering and heatmap of 5 immune cell clusters, 6 gene sets, 10 genes, 7 cytokines and 2 clinical parameters for the asymptomatic and presymptomatic subjects. Z-score normalization was applied for each variable or parameter. **d** PCA of the 5 immune cell clusters, 6 gene sets, 10 genes, 7 cytokines and 2 clinical parameters for the asymptomatic and presymptomatic subjects. Each dot represents a subject, colored by disease status. **e** Integrated unsupervised hierarchical clustering and heatmap of 4 immune cell clusters (T04, M02, M03 and M04), 2 gene sets (Innate immunity: Macrophage infiltration; GO: Osteoclast differentiation, 5 genes (*FAM110B*, *PAK3*, *PPP1R17*, *UTS2* and *CCR5*), and 1 cytokine (STC1) for the asymptomatic and presymptomatic subjects. **f** Contributions of the variables to Dim−1 and Dim−2. The variables including 4 immune cell clusters (T04, M02, M03 and M04), 2 gene sets (Innate immunity: Macrophage infiltration; GO: Osteoclast differentiation, 5 genes (*FAM110B*, *PAK3*, *PPP1R17*, *UTS2* and *CCR5*), and 1 cytokine (STC1).
