# Supplementary material for: Distinct immune signatures discriminate between asymptomatic and presymptomatic SARS-CoV-2pos subjects
Source: Cell Res. 2021 Sep 24;31(11):1148–62. doi: 10.1038/s41422-021-00562-1 (PMC8461439; doi:10.1038/s41422-021-00562-1)
Supplement: Supplementary file 9 — Supplementary information, Figure S9 [file 41422_2021_562_MOESM9_ESM.pdf]

Supplementary information, Figure S9

a

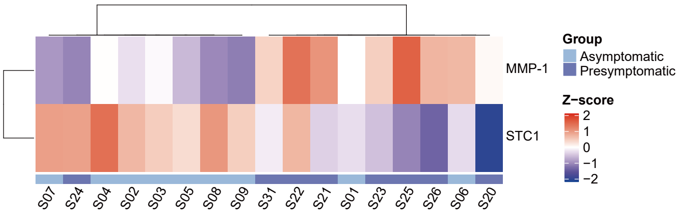

b

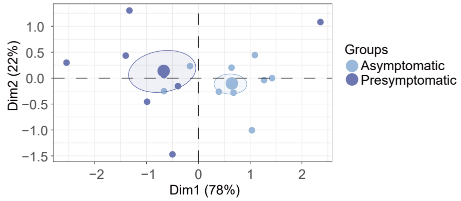

c

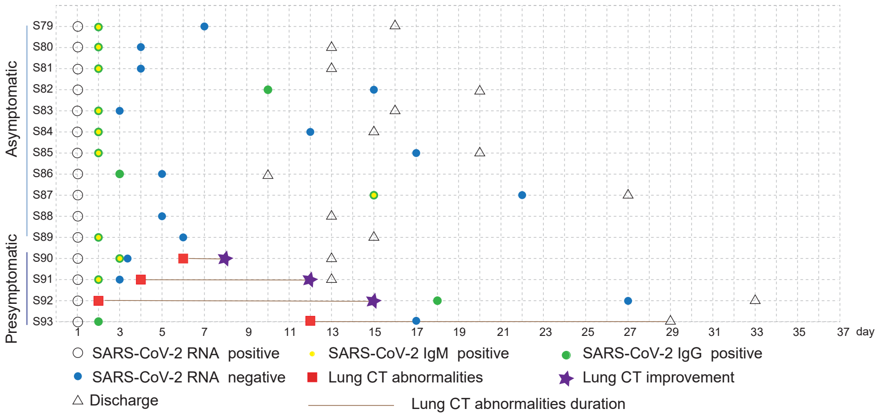

d

	SARS-CoV-2 infections	
	Asymptomatic subjects (n=11)	Presymptomatic subjects (n=4)
The duration of viral shedding, days	4.00 (3.00-13.00)	9.00 (2.00-16.00)
Days from admission to appearance of pneumonia imaging findings on chest CT or MRI (the incubation period), days	N/A	2.50 (1.00-8.50)
Hospitalization days	12.00 (10.00-16.00)	18.00 (10.00-29.00)
SARS-CoV-2 IgM or IgG in serum, no./total no. (%)		
Positive	10/11 (90.9)	4/4(100)
Negative	1/11 (9.1)	0/4 (0)

Data are shown as median (IQR) unless otherwise noted.

e

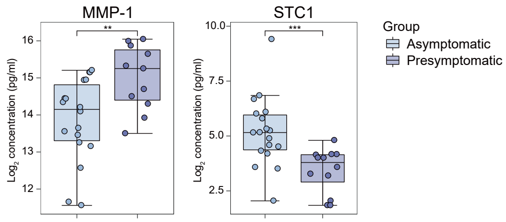

f

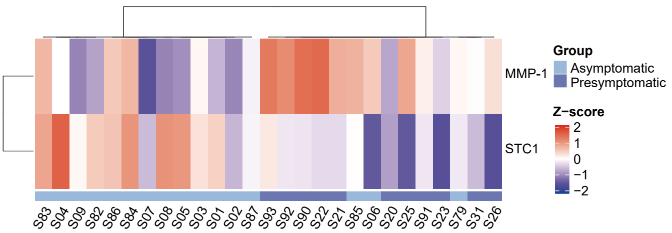

Supplementary information, Figure S9. The prediction power of the two plasma proteins at SSIS.

a Heatmap depicting the expression of MMP-1 and STC1 measured by Olink in the asymptomatic and presymptomatic subjects of the Cohort 1. **b** PCA of the expression of MMP-1 and STC1 measured by Olink in the asymptomatic and presymptomatic subjects of the Cohort 1. Each dot represents a subject, colored by disease status. **c** The summarized clinical histories of the asymptomatic and presymptomatic subjects of the Cohort 2. **d** The clinical characteristics of the 11 asymptomatic and 4 presymptomatic subjects of the Cohort 2. **e** Concentration of plasma MMP-1 and STC1 measured by ELISA in the asymptomatic and presymptomatic subjects of the combined Cohort 1 and Cohort 2. **f** Heatmap depicting the expression of MMP-1 and STC1 measured by ELISA in the asymptomatic and presymptomatic subjects of the combined Cohort 1 and Cohort 2. Significance was determined by unpaired Wilcoxon test. **$p < 0.01$, ***$p < 0.001$.
